# Embryonic and postembryonic development of the ornamental twin‐tail goldfish

**DOI:** 10.1002/dvdy.15

**Published:** 2019-02-19

**Authors:** Ing‐Jia Li, Shu‐Hua Lee, Gembu Abe, Kinya G. Ota

**Affiliations:** ^1^ Laboratory of Aquatic Zoology, Marine Research Station Institute of Cellular and Organismic Biology, Academia Sinica Yilan, 26242 Taiwan; ^2^ Department of Ecological Developmental Adaptability Life Sciences Graduate School of Life Sciences, Tohoku University Sendai 980‐8578 Japan

**Keywords:** Artificial selection, domestication, chordin, dorsal‐ventral patterning

## Abstract

**Background:**

Twin‐tail ornamental goldfish have “bifurcated median fins,” a peculiar morphology known to be caused by a mutation in the *chdA* gene. However, several ambiguities regarding the development of the phenotype remain due to a paucity of detailed observations covering the entire developmental timeframe.

**Results:**

Here, we report a detailed comparative description of embryonic and postembryonic development for two representative twin‐tail ornamental goldfish strains and single‐tail common goldfish. Our observations reveal a polymorphic developmental process for bifurcated median fins; disrupted axial skeletal development at early larval stages; and modified bilateral location of the pelvic fin.

**Conclusions:**

Variations in development of bifurcated median fins and disrupted axial skeletal patterns reflect how artificial selection for adult morphological features influenced molecular developmental mechanisms during the domestication of twin‐tail ornamental goldfish. The polymorphic appearance of bifurcated median fins also implies that, unlike previously proposed hypotheses, the development of these structures is controlled by molecular mechanisms independent of those acting on the pelvic fin. Our present findings will facilitate further study of how modifications of preexisting developmental systems may contribute to novel morphological features. *Developmental Dynamics* 248:251–283, 2019. © 2019 The Authors. Developmental Dynamics published by Wiley Periodicals, Inc. on behalf of American Association of Anatomists.

## Key Findings

- This is the first complete study to describe the developmental progression of twin‐tail goldfish. Disrupted axial skeletal morphology in adults develops from a modified osteogenesis process in vertebral elements. The developmental processes for not only the caudal and anal fins, but also pelvic fin, were changed by artificial selection in twin‐tail goldfish. Polymorphic anal and caudal fin development suggested that in addition to the mutation in the chdA gene, other relevant mutations have accumulated in the twin‐tail goldfish. Our developmental observations pave the way to study how the pre‐existing developmental systems were modified by selective pressure for the formation of a novel morphology.

## Introduction

Ornamental goldfish strains exhibit highly divergent morphologies (Ota and Abe, 2016), and among the variations, a twin‐tail with bifurcated axial skeleton represents one of the most unique morphological features (Smartt, 2001; Ota and Abe, 2016). In fact, no other genetically fixed twin‐tail vertebrate animal has been found in natural or domesticated populations thus far (Korschelt, 1907; Ota and Abe, 2016). Our recent molecular developmental genetics study revealed that this exceptional morphological feature is caused by a mutation in the *chordin* gene, which is known as an important player in dorsal‐ventral patterning (Abe et al., 2014). Intensive molecular cloning in several different types of ornamental twin‐tail goldfish strains further revealed that the goldfish genome contains two paralogous *chordin* genes (*chdA* and *chdB*), and the *chdA* gene has a stop codon mutant allele (*chdA* ^*E127X*^) that causes the twin‐tail phenotype. Through the analysis of embryonic gene‐expression patterns, *chdA* and *chdB* appear to be subfunctionalized (Abe *et al*., 2014), suggesting that although the *chdA* ^*E127X/E127X*^ genotype causes reductions of dorsal tissue and simultaneous increases in ventral tissue of goldfish early embryos, the *chdB* gene may partially compensate for the lost function of *chdA*. Consequently, the twin‐tail goldfish exhibits a sufficient survival rate for domestic maintenance, unlike conventional *chordin* gene–depleted vertebrates (Bachiller et al., 2003; Fisher and Halpern, 1999; Takashima et al., 2007).

This relatively high survival rate for *chdA*‐mutant goldfish might increase the chances of establishing various types of stable ornamental twin‐tail goldfish strains (Ota and Abe, 2016; Smartt, 2001). These established variations in ornamental goldfish strains intrigued early and modern researchers alike (Watase, 1887; Bateson, 1894; Koh, 1931,1932; Asano and Kubo, 1972; Smartt, 2001). For example, it has been shown that the laterally bifurcated caudal fin has several different variations in the shapes and numbers of internal and external skeletal elements (Watase, 1887; Bateson, 1894; Smartt, 2001; Ota and Abe, 2016). Various morphologies of bifurcated anal and caudal fins in the ornamental goldfish were also reported by early researchers (Watase, 1887; Bateson, 1894; Smartt, 2001; Ota and Abe, 2016) and used as an example of discontinuous variation by Bateson (1894). Moreover, the extremely short and globular morphologies of ornamental goldfish strains provided a motivation for classical anatomical researchers to investigate internal skeletal morphologies (Koh, 1931, 1932; Asano and Kubo, 1972). It is certain that these well described morphological variations provide a strong basis to gather empirical evidence for further understanding of how morphological variations can be produced from artificial selection via the modification of developmental mechanisms (Ota and Abe, 2016). However, our understanding of twin‐tail ornamental goldfish development is still limited due to a paucity of information showing the details of developmental progression.

Several early studies have reported details of goldfish development (Watase, 1887; Khan, 1929; Battle, 1940; Hervey and Hems, 1948; Li et al., 1959; Kajishima, 1960; Yamaha et al., 1999; Nagai et al., 2001; Otani et al., 2002). However, these early reports lack information about the morphology of the parents, presumably because the morphological variations in the parents were beyond the research focus. Although we also reported embryonic and postembryonic developmental processes in the single‐tail common goldfish (Tsai et al., 2013; Li et al., 2015), our previous studies did not address twin‐tail goldfish.

Thus, in this study, we observed the embryonic and postembryonic development of twin‐tail ornamental goldfish strains with reference to fin and postcranial skeletal morphology. These studies revealed differences and similarities between the twin‐tail ornamental goldfish and single‐tail common goldfish in the developmental rate and timing of appearance for morphological features as measured by fluorescence microscopy and histology. Our report provides further insight into how the highly diverged morphology of twin‐tail ornamental goldfish is established by the *chdA* gene mutation and subsequent additional mutations under artificial selection, providing a platform for further studies on the evolutionary developmental biology of vertebrates.

## Results

### Morphological Variations of Parent Goldfish

**Figure 1:**
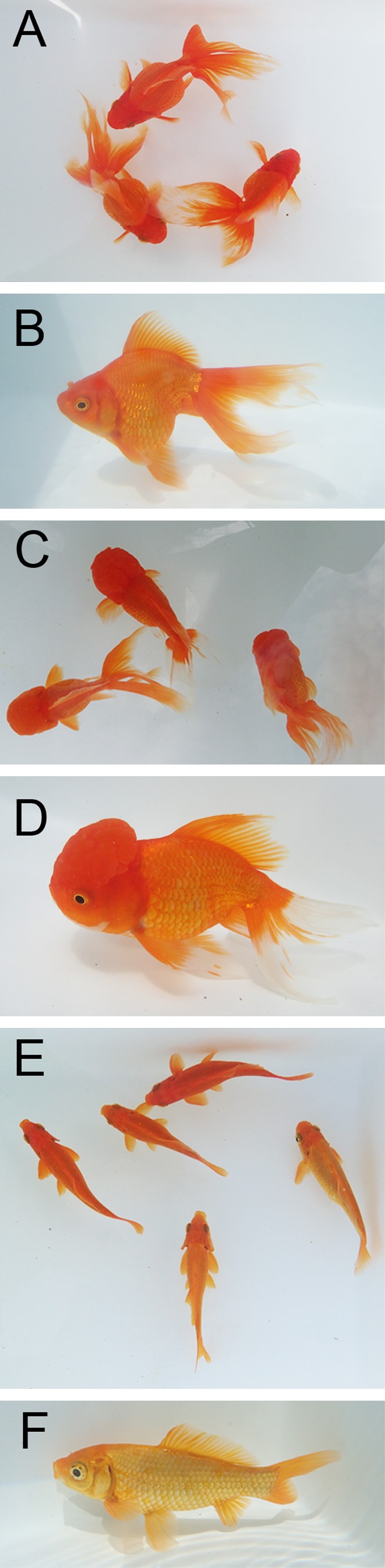
Representative adult morphology of twin‐tail ornamental and single‐tail common goldfish strains. **A:** Dorsal view of *Ryukin* adult fish. **B:** Oblique lateral view of *Ryukin* adult fish. **C:** Dorsal view of *Oranda* strain adults. **D:** Oblique lateral view of *Oranda* adult fish. **E:** Dorsal view of the single‐tail common goldfish adult. **F:** Oblique lateral view of the single‐tail common goldfish. All of the pictured goldfish are approximately 12 cm standard length.

We examined two different ornamental goldfish strains, *Ryukin* and *Oranda shishigashira* (*Oranda*), in this study, since both are empirically known as easy to maintain (Abe et al., 2014) (Fig. 1A–D). Although the *Oranda* strain is distinguished from the *Ryukin* strain by the presence of well developed warty growth around the cranium, the two strains share similar morphological features, including a bifurcated caudal fin, a globular body, and the presence of a dorsal fin (Smartt, 2001; Abe et al., 2014; Ota and Abe, 2016) (Fig. 1B,D). The acquired data and images of twin‐tail goldfish were compared with previous reports of single‐tail common goldfish (Tsai et al., 2013; Li et al., 2015). Moreover, we obtained additional data from a single‐tail common goldfish strain, which has a slender body and short tail, for direct comparison (Fig. 1E,F). We obtained progenies between April and July in 2016 and 2017. For the detailed observations of development, five progeny populations were derived from the same clutches and were designated by ID numbers as follows: #2017‐0307‐RY, #2017‐0320‐RY, #2017‐0425‐OR, #20170508‐OR, and #20170420‐Single (eight‐digit numbers indicate spawning date; RY: *Ryukin*, OR: *Oranda*, Single: single‐tail common goldfish).

### Life‐history Stage and Period Definitions

**Figure 2:**
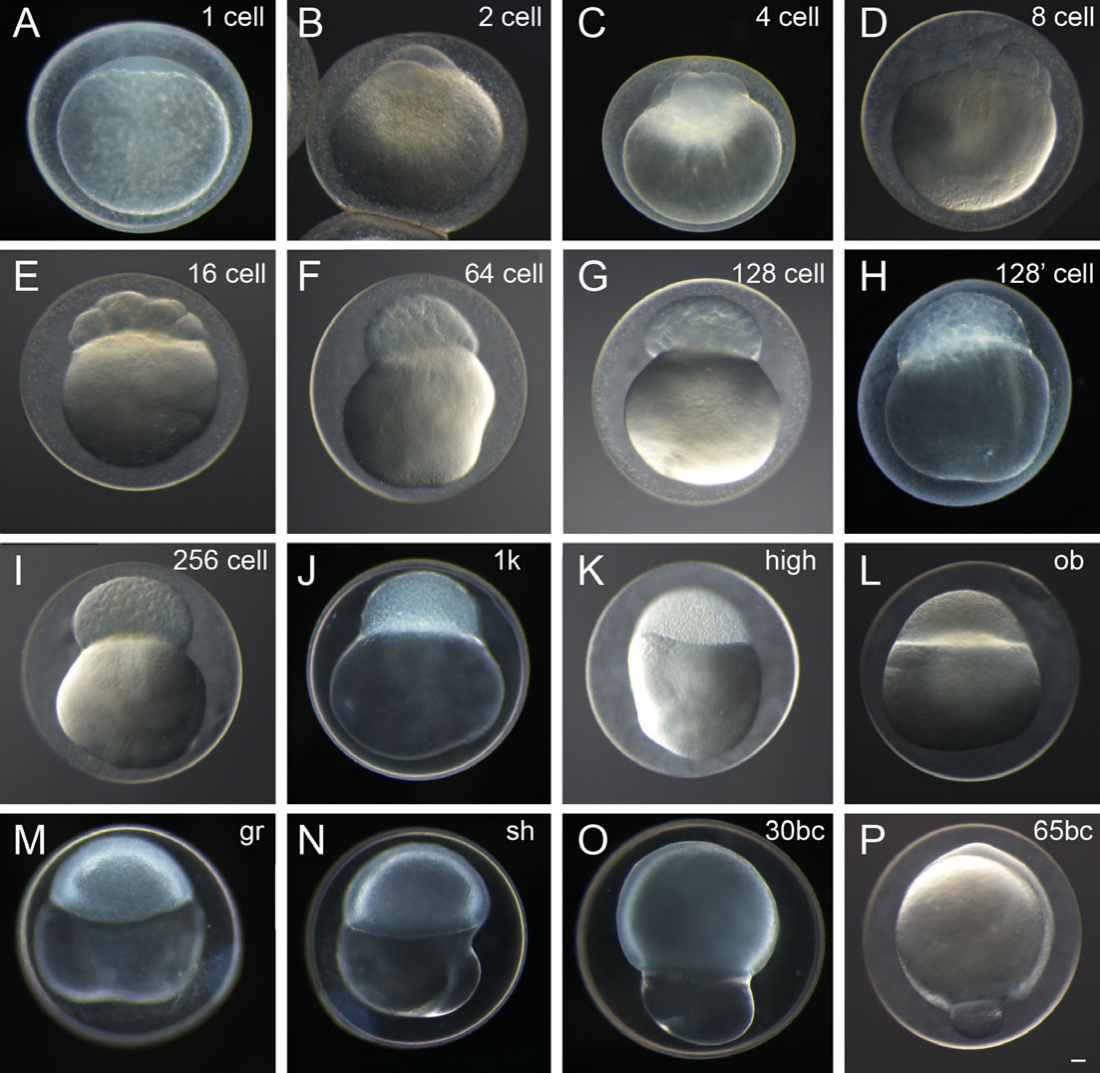
Representative fertilization to cleavage, blastula, and gastrula periods of twin‐tail goldfish embryos. **A:** Zygote stage. **B–F:** Cleavage stages. **G–L:** Blastula stages. **M–P:** Gastrula stages. Designations in the upper right corner of each panel indicate stage. Panel H is labeled as 128’ cell and shows an intermediate stage between 128‐cell and 256‐cell stages. Panels A–B,D–P are *Ryukin* embryos. Panel C shows an embryo of the *Oranda* strain. bc, blastopore closure; gr, germ ring; ob, oblong; sh, shield. Scale bar P = 0.1 mm. All panels are shown at the same magnification.

**Figure 3:**
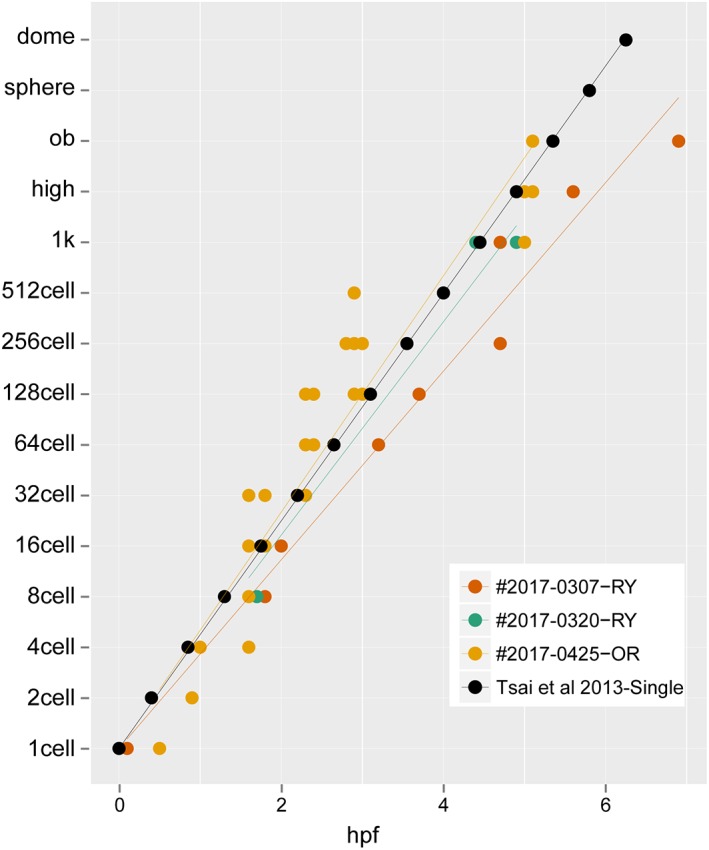
Rates of development for embryos from zygote to early gastrula**.** The data were sampled from 87 points, consisting of 17, 9, and 61 points from #2017‐0307‐RY, #2017‐0320‐RY, and #2017‐0425‐OR clutches, respectively. The black circles indicate the rate of development for single‐tail goldfish reported in Tsai et al., 2013.

**Figure 4:**
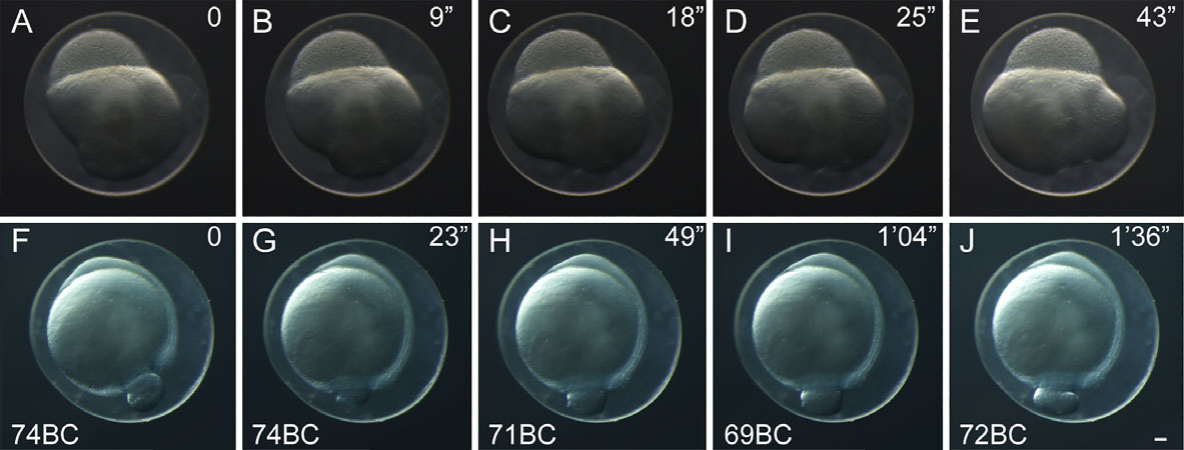
Time‐lapse images of twin‐tail goldfish embryos. Individual time‐lapse images from a representative blastula‐period embryo (**A–E**) and gastrula‐period embryo (**F–J**). The embryos were incubated at 24°C. Lapsed times from the initiation of imaging are indicated at the upper right corner of each panel. Photos of embryos from fertilized eggs of *Ryukin*‐strain parents. Gastrula‐period embryos are indicated as blastopore closure (BC) in the lower left corner. Scale bar J = 0.1 mm. All embryos were photographed at the same magnification.

**Figure 5:**
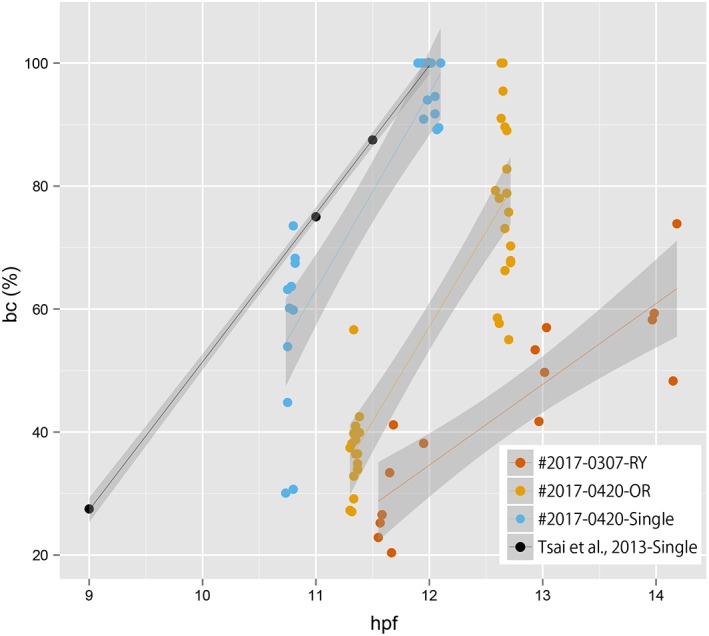
Relationship between the size of blastopore and hours after postfertilization. The data were sampled from 73 points, consisting of 15, 36, and 22 points from #2017‐0307RY, #2017‐0425‐OR, and #2017‐0420‐Single clutches, respectively. Points derived from different clutches are indicated by the different colors. The gray area with regression lines indicates the 95% confidence interval. The black circles indicate the rate of development for single‐tail goldfish reported in Tsai et al., 2013.

**Figure 6:**
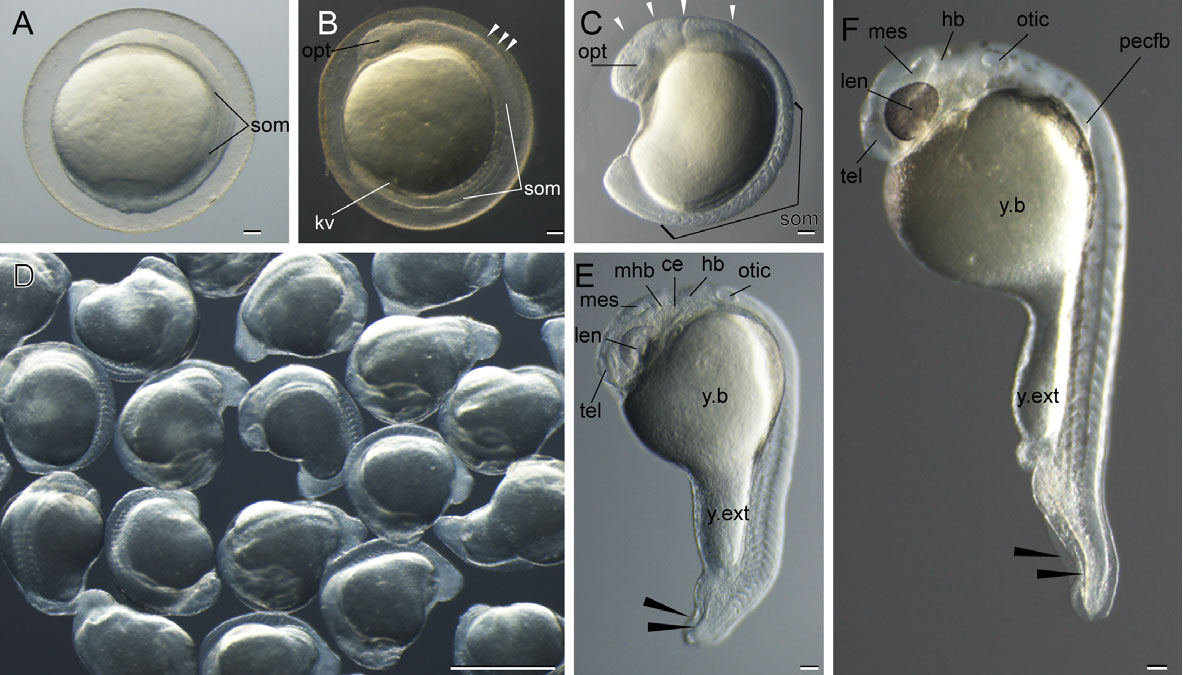
Representative segmentation to pharyngular‐period goldfish embryos. **A:** Four‐somite stage. **B:** Ten‐somite stage. **C:** Eighteen‐somite stage. **D:** Seventeen‐ to nineteen‐somite‐stage embryos. **E:** Twenty‐five‐somite stage. **F:** Pharyngular stage (34% Otic vesicle closure (OVC)). Black and white arrowheads indicate bifurcated fin fold and divisions of the brain rudiment, respectively. Embryos in panels A,E,F are derived from *Ryukin*‐strain parents, and embryos in the other panels are derived from *Oranda*‐strain parents. ce, cerebellum; hb, hindbrain; kv, Kupffer's vesicle; len, lens; mes, mesencephalon; mhb, midbrain‐hindbrain boundary; opt, optic vesicle; otic, otic vesicle; pecfb, pectoral fin bud; som, somite; tel, telencephalon; y.b, yolk ball; y.ext, yolk extension. Scale bars A–C,E,F = 0.1 mm. Scale bar D = 1 mm.

**Figure 7:**
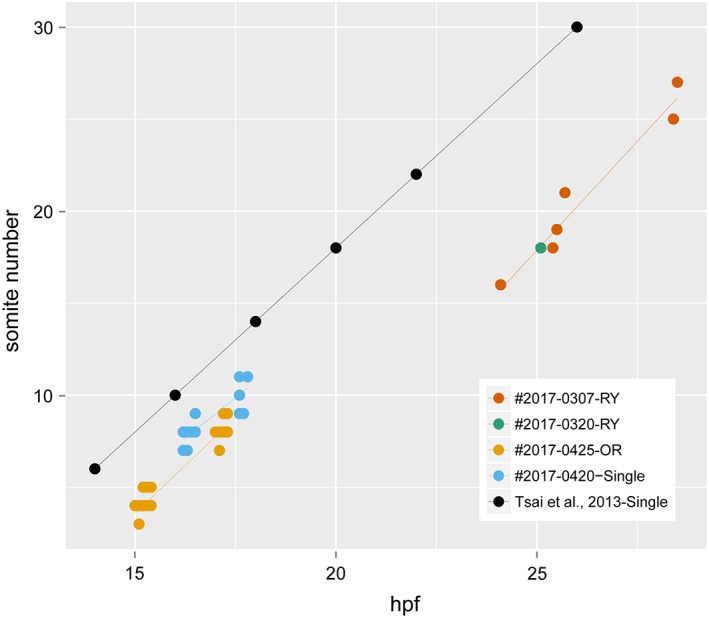
Rates of development for embryos at the segmentation period. The data were sampled from 70 points, consisting of 8, 1, 33, and 28 points from #2017‐0307‐RY, #2017‐0320‐RY, #2017‐0425‐OR, and #2017‐0420‐Single, respectively. The regression line for the developmental rate of *Ryukin* progenies was estimated from the plots of #2017‐0307‐RY and #2017‐0320‐RY. The black circles indicate the rate of development for single‐tail goldfish progenies (Tsai et al., 2013).

**Figure 8:**
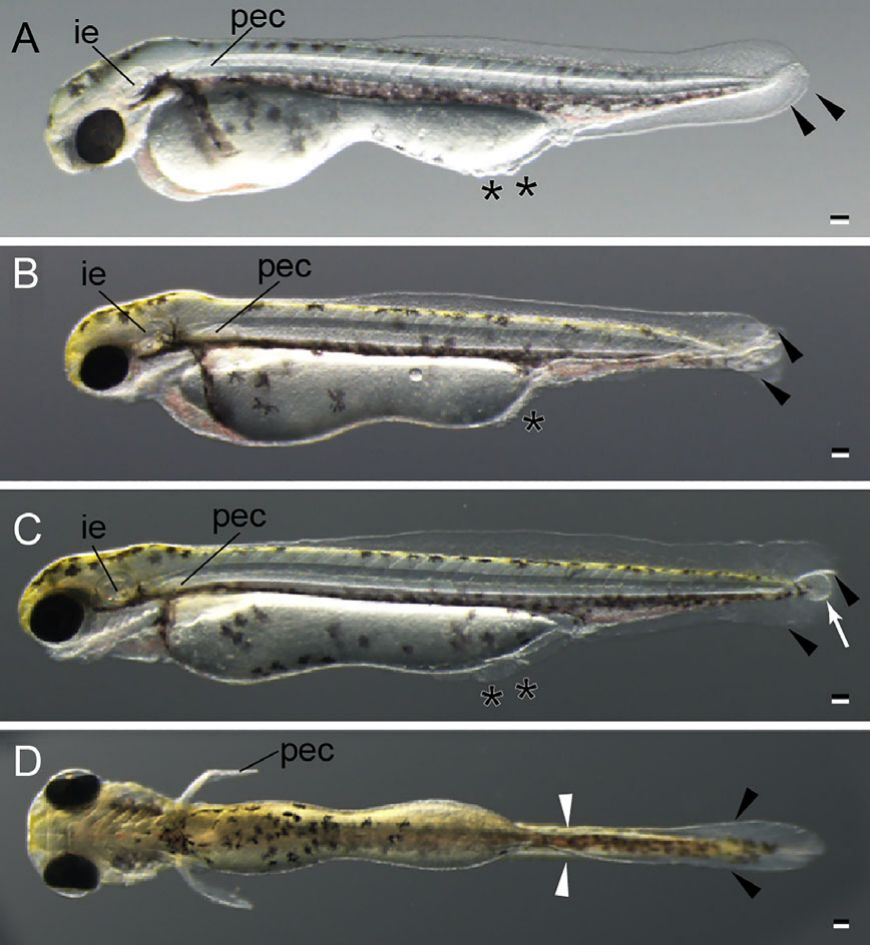
Hatching stage of twin‐tail goldfish larvae. **A:** Lateral view of long pec stage. **B:** Lateral view of pec fin stage. **C:** Lateral view of protruding‐mouth stage. **D:** Ventral view of protruding‐mouth stage. All larvae were derived from *Ryukin*‐strain parents. Black arrowheads and asterisks indicate bifurcated fin fold and malformed fin fold. White arrow indicates edema; white arrowheads indicate the edge of the bifurcated fin fold near the end of the yolk. ie, inner ear; pec, pectoral fin. Scale bars = 0.1 mm.

**Figure 9:**
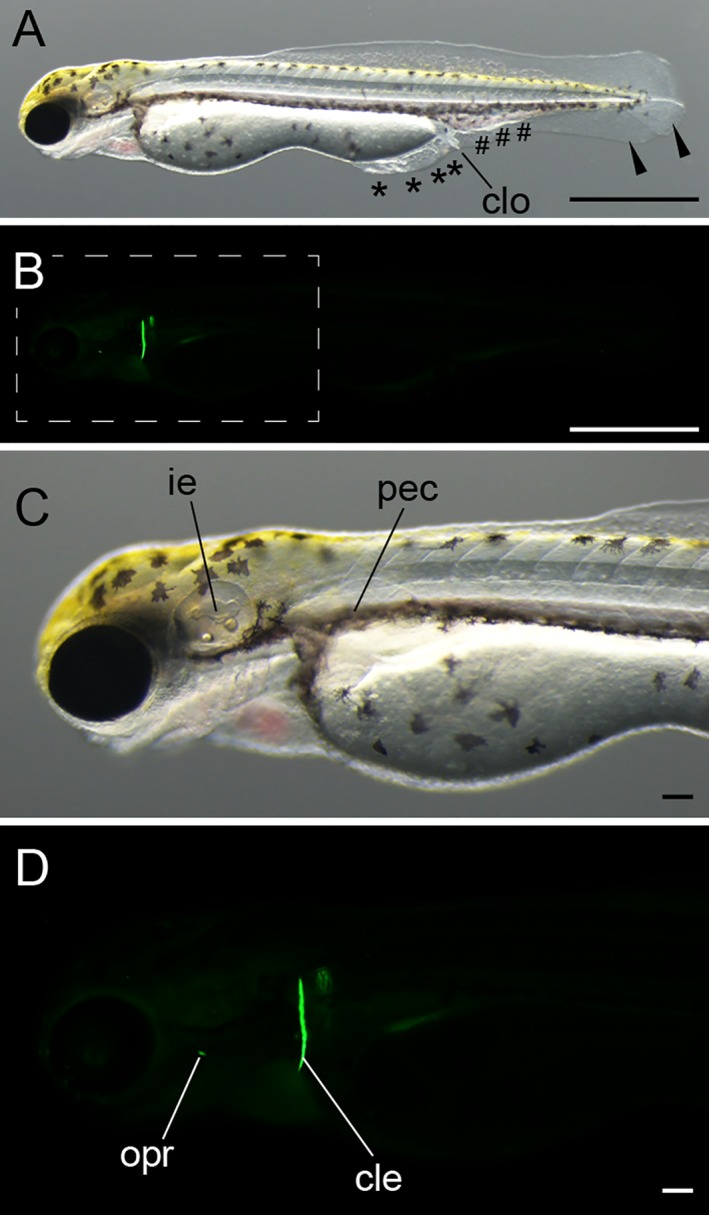
Protruding mouth‐stage larva. **A:** Lateral light microscopic view of the whole body. Black arrowheads indicate bifurcated fin fold; black asterisks indicate malformed fin fold; black pound signs (#) indicate enlarged blood island. **B:** Lateral view of calcein‐stained fluorescence. **C:** Magnified view of panel A. **D:** Magnified view of the boxed region in panel B. The pictured larva was derived from *Ryukin* parents. cle, cleithrum; clo, cloaca; ie, inner ear; opr, opercular pec, pectoral fin. Scale bars A,B = 1 mm. Scale bars C,D = 0.1 mm.

**Figure 10:**
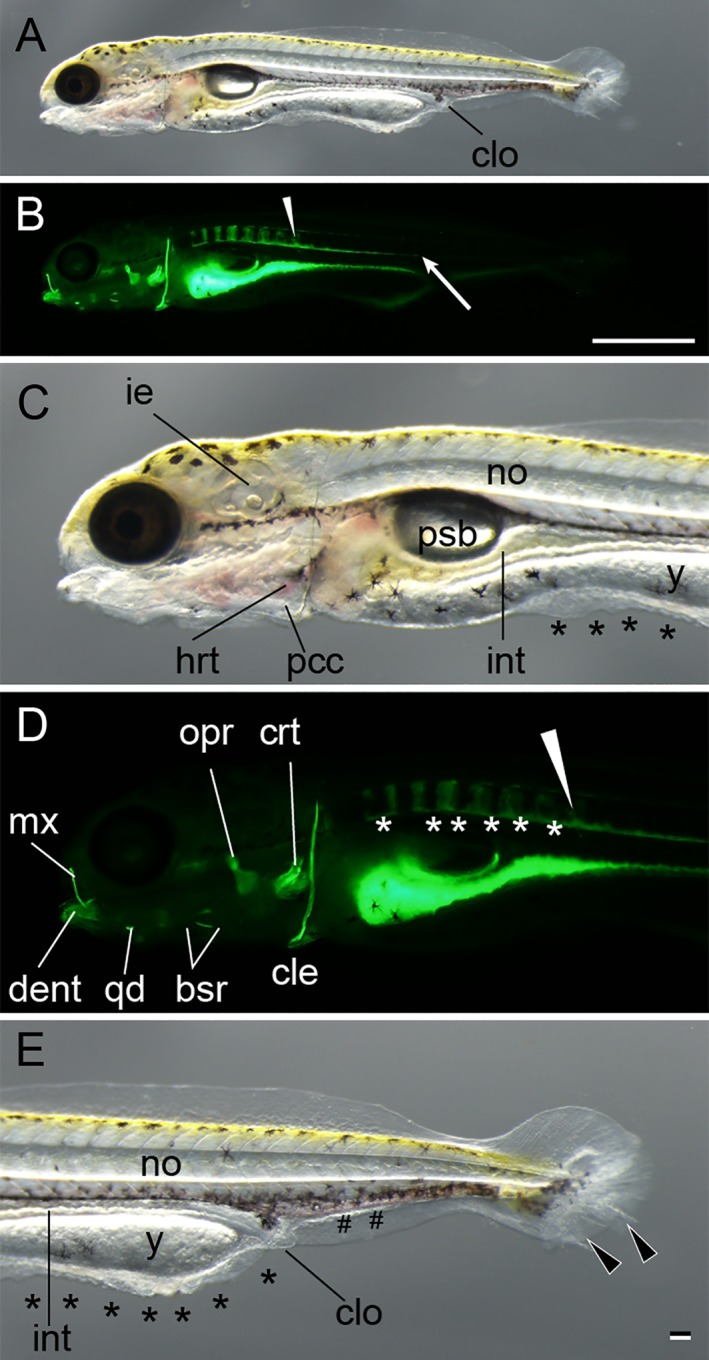
Posterior swim bladder stage. **A–E:** Lateral views of a larva from *Ryukin* parents. Panels A–E are light microscopic views of the entire body, calcein‐stained fluorescence views of the entire body, magnified views of the anterior region of A, magnified views of the anterior region B, and magnified views of caudal region of A, respectively. Bifurcated caudal fins are indicated by the black arrowheads. Malformed fin folds are marked by black asterisks; enlarged blood vessels are marked by pound signs (#). White arrowheads mark the most posterior calcified vertebral body. White asterisks indicate calcein‐stained area between calcified vertebral elements. White arrow shows the posterior end of unsegmented calcein‐positive tissues on the ventral side of the notochord. bsr, branchiostegal rays; cle, cleithrum; clo, cloaca; crt, ceratobranchial; dent, dentary; hrt, heart; mx, maxilla; ie, inner ear; int, intestine; no, notochord; opr, opercular; pcc, pericardial cavity; psb, posterior swim bladder; qd, quadrate; y, yolk. Scale bar B = 1 mm. Scale bar E = 0.1 mm. Panels of the entire larva view (A,B), panels of the magnified view (C–E) were photographed at the same magnification.

**Figure 11:**
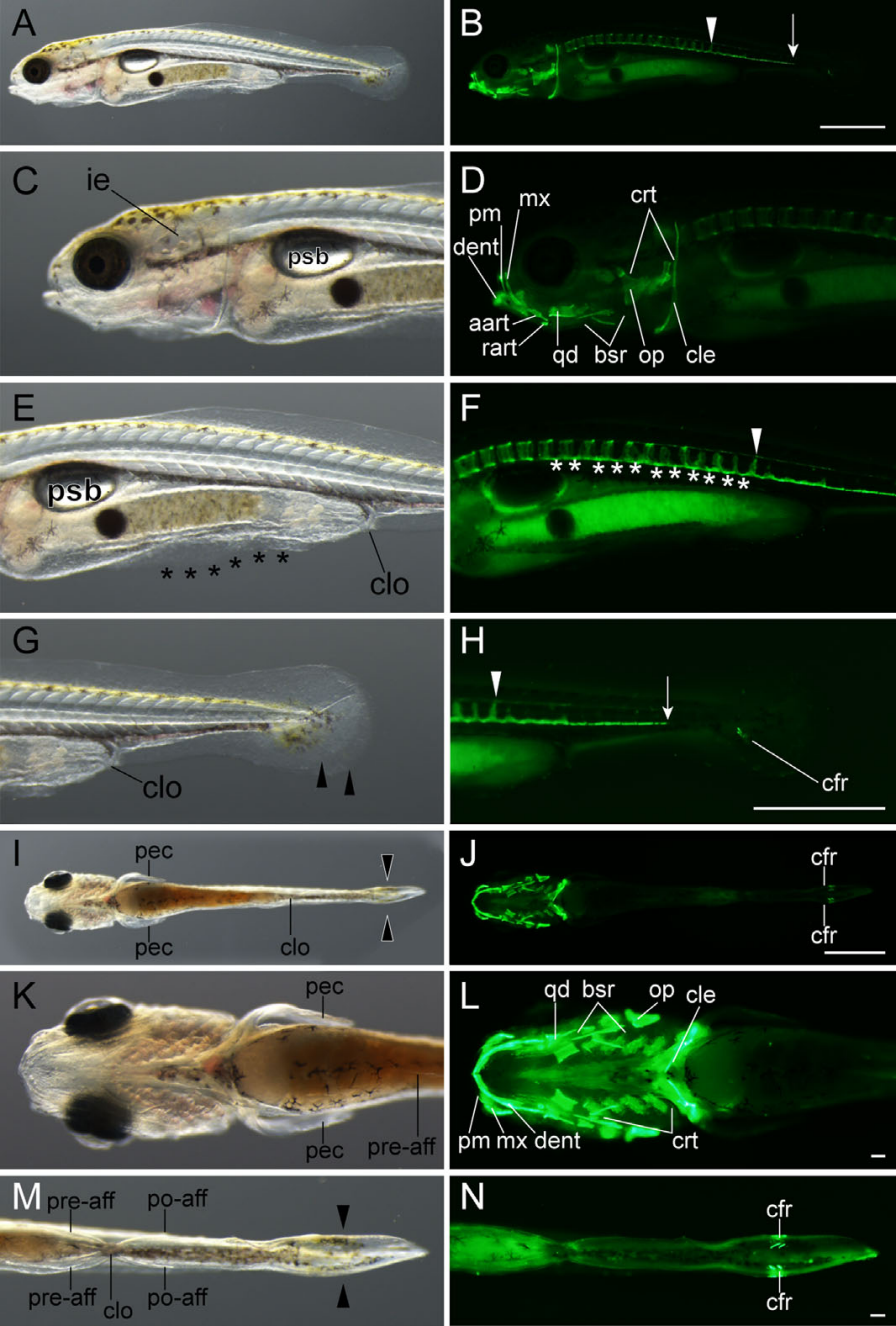
Early caudal fin ray–stage larvae. **A–H:** Lateral views of *Ryukin*‐strain larva at two‐caudal‐fin‐ray stage. **I–N:** Ventral views of *Ryukin*‐strain larva. Left and right columns show light and calcein‐stained fluorescence microscopic images. Second‐, third‐, and fourth‐row panels are magnified views of panels in the first row. Panels in the sixth and seventh rows are magnified from panels in the fifth row. White arrows, arrowheads, and asterisks indicate the most posterior part of the calcified notochord, the most posterior calcified centrum, and calcified notochordal regions between centra, respectively. Black asterisks and arrowheads mark bifurcated caudal fin and malformed pre‐anal fin fold. aart, anguloarticular; bsr, branchiostegal rays; cfr, caudal fin ray; cle, cleithrum; clo, cloaca; crt, ceratobranchial; dent, dentary; ie, inner ear; mx, maxilla; op, opercular; pec, pectoral fin; pm, premaxilla; po‐aff, post‐anal fin; pre‐aff, pre‐anal fin; psb, posterior swim bladder; qd, quadrate; rart, retroarticular. Scale bars B,H = 1 mm. Scale bars = 0.1 mm. Panels in the first row (A,B), second to fourth rows (C–H), fifth row (I,J), and sixth and seventh rows (K–N) are shown at the same magnifications.

**Figure 12:**
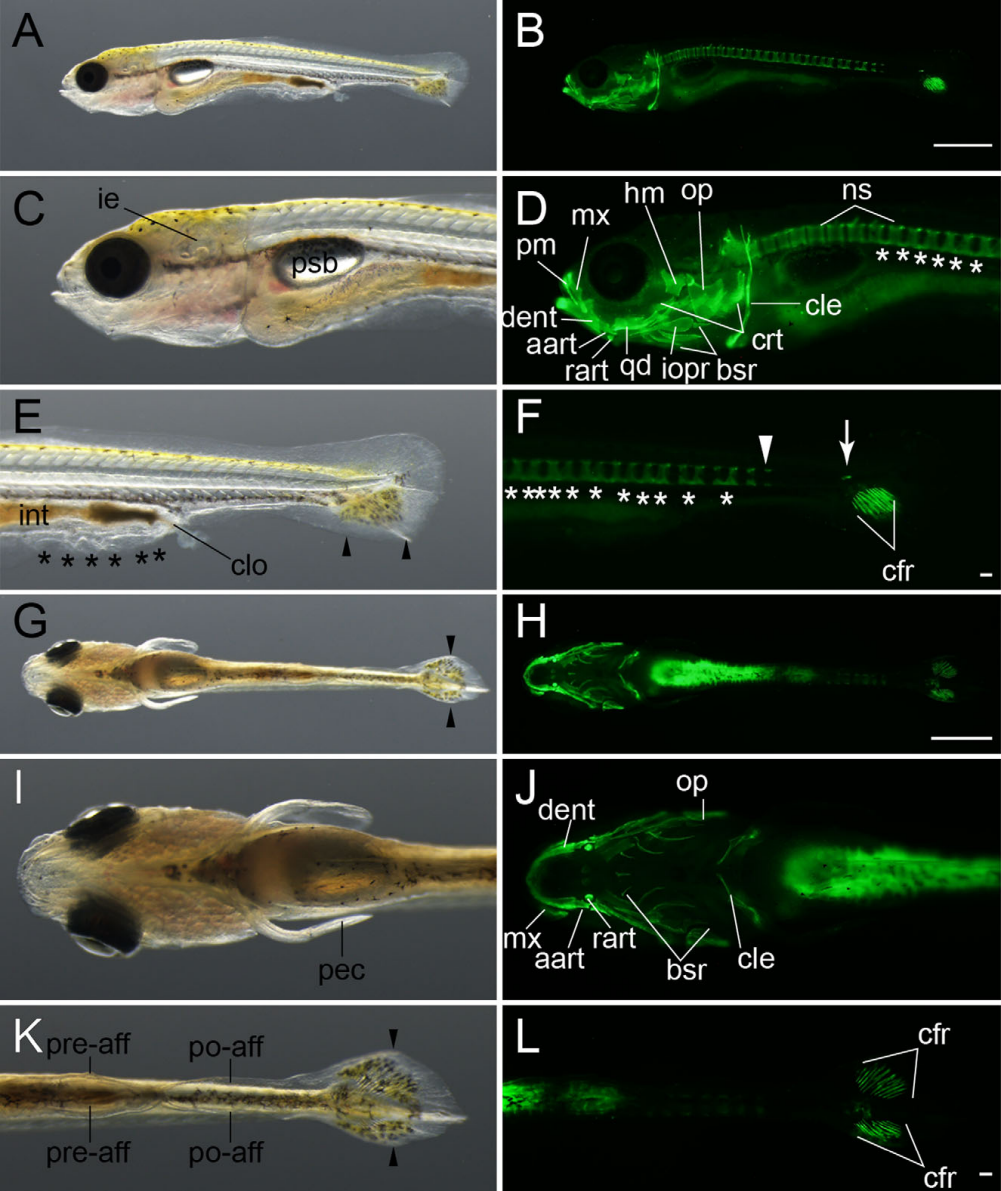
Late caudal fin ray–stage larvae. **A–F:** Lateral views of an eight caudal fin ray–stage larva from *Ryukin* parents. **G–L:** Ventral view of an eleven caudal fin ray–stage *Ryukin* progeny. Left and right columns show light and calcein‐stained fluorescein microscopic images. Black asterisks and arrowheads indicate bifurcated caudal fin and malformed pre‐anal fin fold. White asterisks and arrowheads mark ectopically calcified notochordal region and the most posterior calcified centrum. White arrow shows calcified tissue at the level of the flexed notochord. aart, anguloarticular, bsr, branchiostegal rays; cfr, caudal fin rays; cle, cleithrum; clo, cloaca; crt, ceratobranchial; dent, dentary; hm, hyomandibular; ie, inner ear; int, intestine; iopr, interopercular; mx, maxilla; ns, neural spine; op, opercular; pec, pectoral fin; pm, premaxilla; po‐aff; post‐anal fin fold; pre‐aff, pre‐anal fin fold; psb, posterior swim bladder; qd, quadrate; rart, retroarticular. Scale bars B,H = 1 mm. Scale bars F,L = 0.1 mm. Panels of the first row (A,B), second and third rows (C–F), fourth row (G,H), and fifth and six rows (I–L) were photographed at the same magnifications.

**Figure 13:**
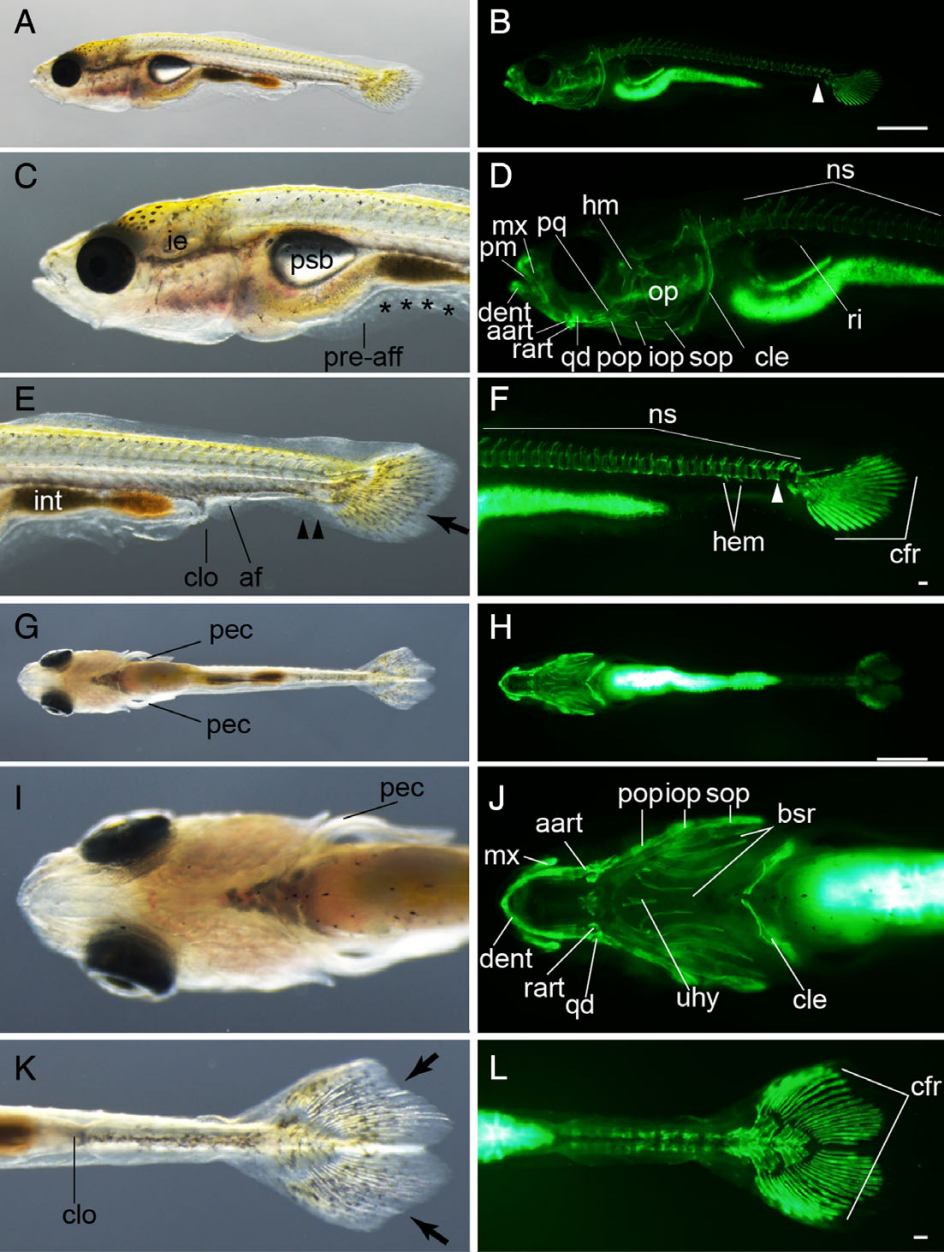
Forked caudal fin–stage larvae. **A–F:** Lateral views of a forked caudal fin–stage larva from *Ryukin* parents. **G–L:** Ventral views of a forked caudal fin–stage *Ryukin* progeny. Left column (A,C,E,G,I,K) and right column (B,D,F,H,J,L) show light and calcein‐stained fluorescent microscopic images. Black arrows, arrowheads, and asterisks indicate the concave point that divides the upper and lower fin lobes, bifurcated caudal fin fold, and malformed pre‐anal fin fold, respectively. White arrowheads show the most posterior calcified centrum. aart, anguloarticular; af, anal fin; bsr, branchiostegal rays; cfr, caudal fin rays; cle, cleithrum; clo, cloaca; dent, dentary; hem, hemal arch; hhyv, hyophyal ventral; hm, hyomandibula; ie, inner ear; int, intestine; iop, interopercular; mx, maxilla; ns, neural spine; op, opercular; pec, pectoral fin; pm, premaxilla; pop, preopercular; pq, palatoquadrate; pre‐aff, pre‐anal fin fold; psb, posterior swim bladder; qd, quadrate; rart, retroarticular; ri, rib; sop, subopercular; uhy, urohyal. Scale bars B,H = 1 mm. Scale bars F,L = 0.1 mm. Panels of the first row (A,B), second and third rows (C–F), fourth row (G,H), and fifth and six rows (I–L) are shown at the same magnifications.

**Figure 14:**
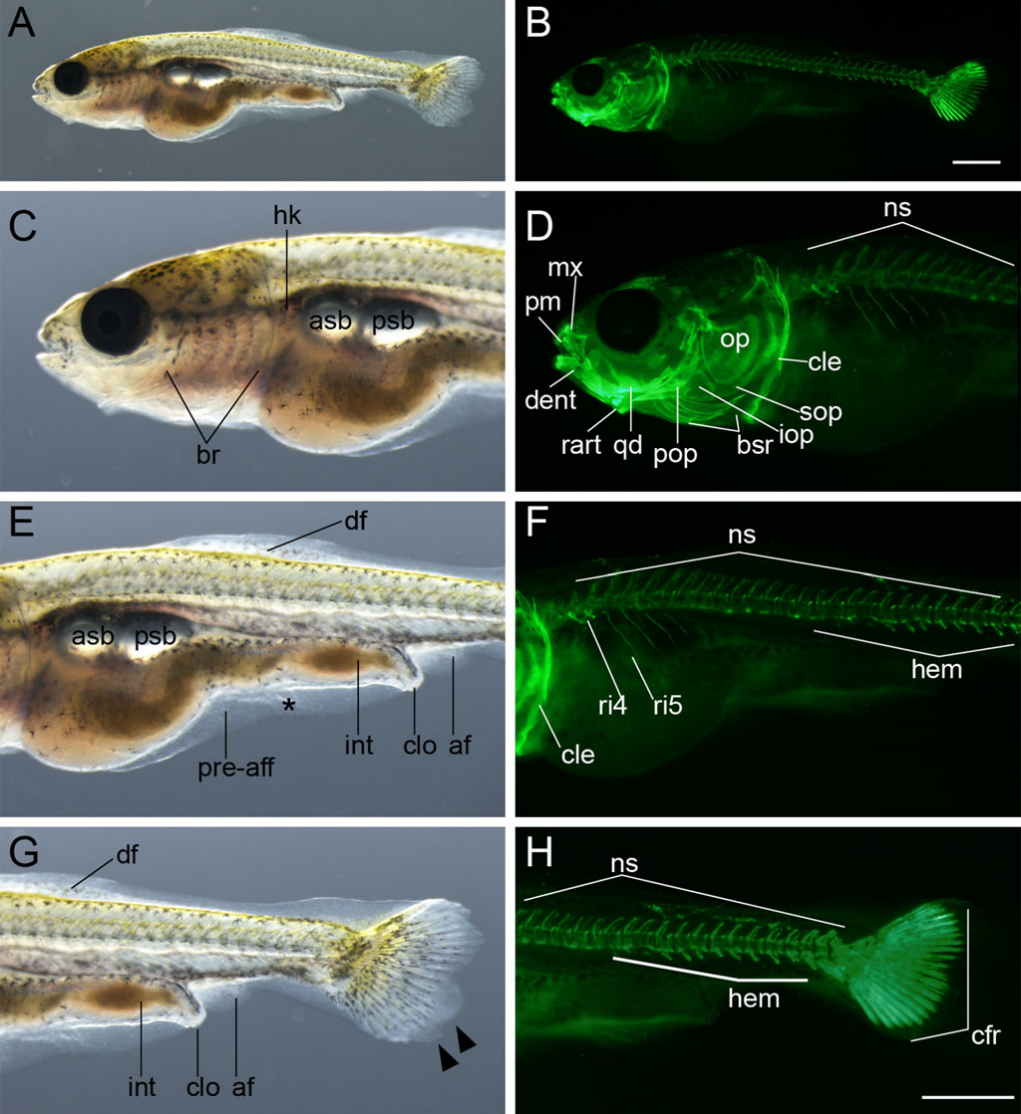
Anterior swim bladder–stage larva. **A,B:** Lateral whole‐body view of larva from *Ryukin* parents. C–H: Magnified views at the anterior region (**C,D**), mid‐trunk region (**E,F**), and caudal region (**G,H**) of A,B. Black asterisk represents malformed pre‐anal fin fold. af, anal fin; asb, anterior swim bladder; br, branchial; bsr, branchiostegal rays; cfr, caudal fin rays; cle, cleithrum; clo, cloaca; dent, dentary; df, dorsal fin; hem, hemal arch; hk, head kidney; int, intestine; iop, interopercular; mx, maxilla; ns, neural spine; op, opercular; pm, premaxilla; pop, preopercular; pre‐aff, pre‐anal fin fold; psb, posterior swim bladder; qd, quadrate; rart, retroarticular; ri, rib; sop, subopercular. Scale bars B,H = 1 mm. Panels of the entire larva view (A,B) and panels of the magnified view (C–H) were photographed at the same magnification.

**Figure 15:**
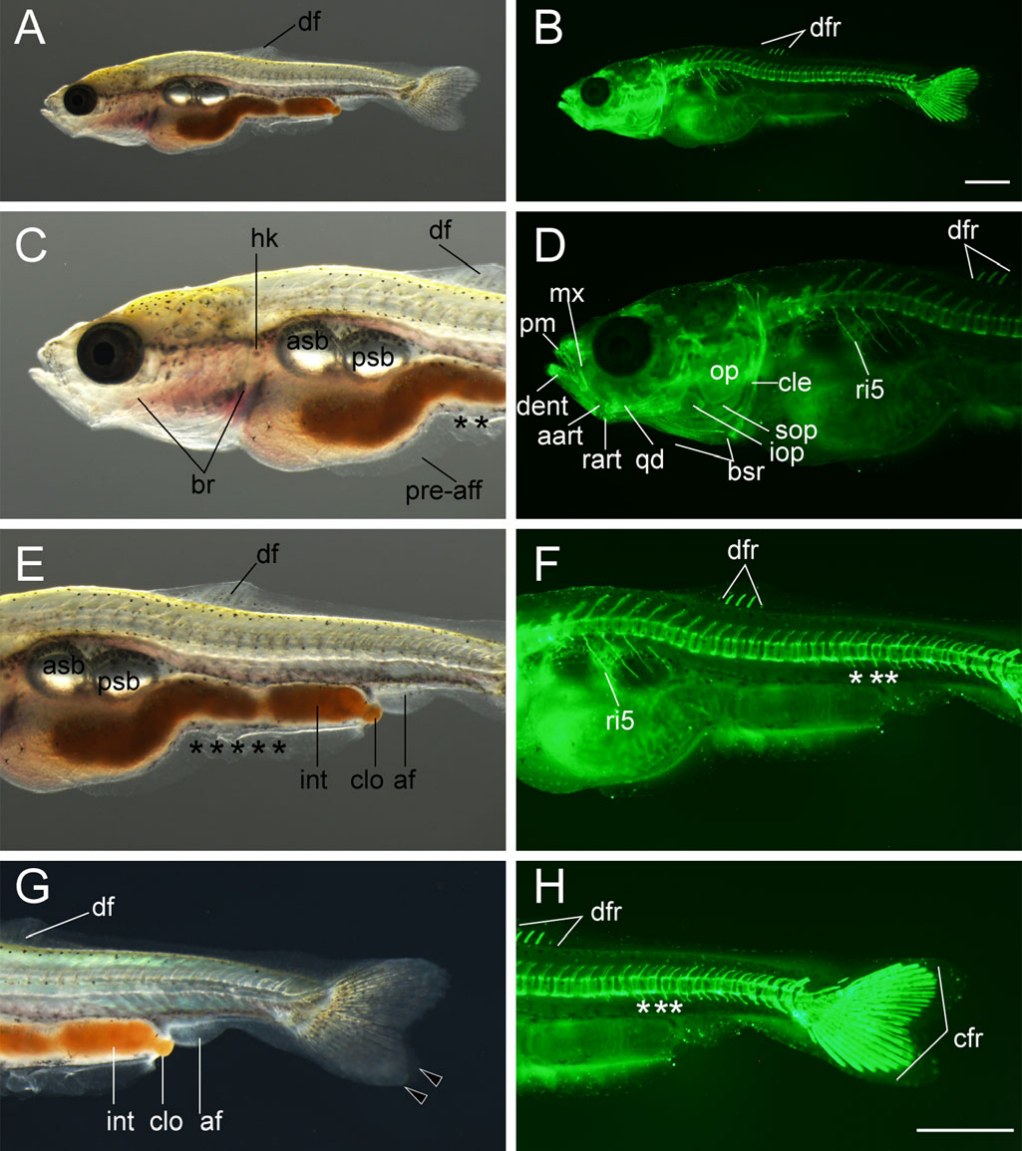
Dorsal fin ray–stage larva. **A,B:** Lateral views of whole body of larva from *Ryukin* parents. C–H: Magnified views of anterior (**C,D**), mid‐trunk (**E,F**), and posterior regions (**G,H**). Black asterisks mark malformed pre‐anal fin fold. Black arrowheads indicate bifurcated caudal fins. White asterisks indicate fused centrum. aart, anguloarticular; af, anal fin; asb, anterior swim bladder; br, branchial; bsr, branchiostegal rays; cfr, caudal fin rays; cle, cleithrum; clo, cloaca; dent, dentary; df, dorsal fin; dfr, dorsal fin rays; hk, head kidney; int, intestine; iop, interopercular; mx, maxilla; op, opercular; pm, premaxilla; pre‐aff, pre‐anal fin fold; psb, posterior swim bladder; qd, quadrate; rart, retroarticular; ri, rib; sop, subopercular. Scale bars B,H = 1 mm. Panels of the entire larva view (A,B) and panels of the magnified view (C–H) were photographed at the same magnification.

**Figure 16:**
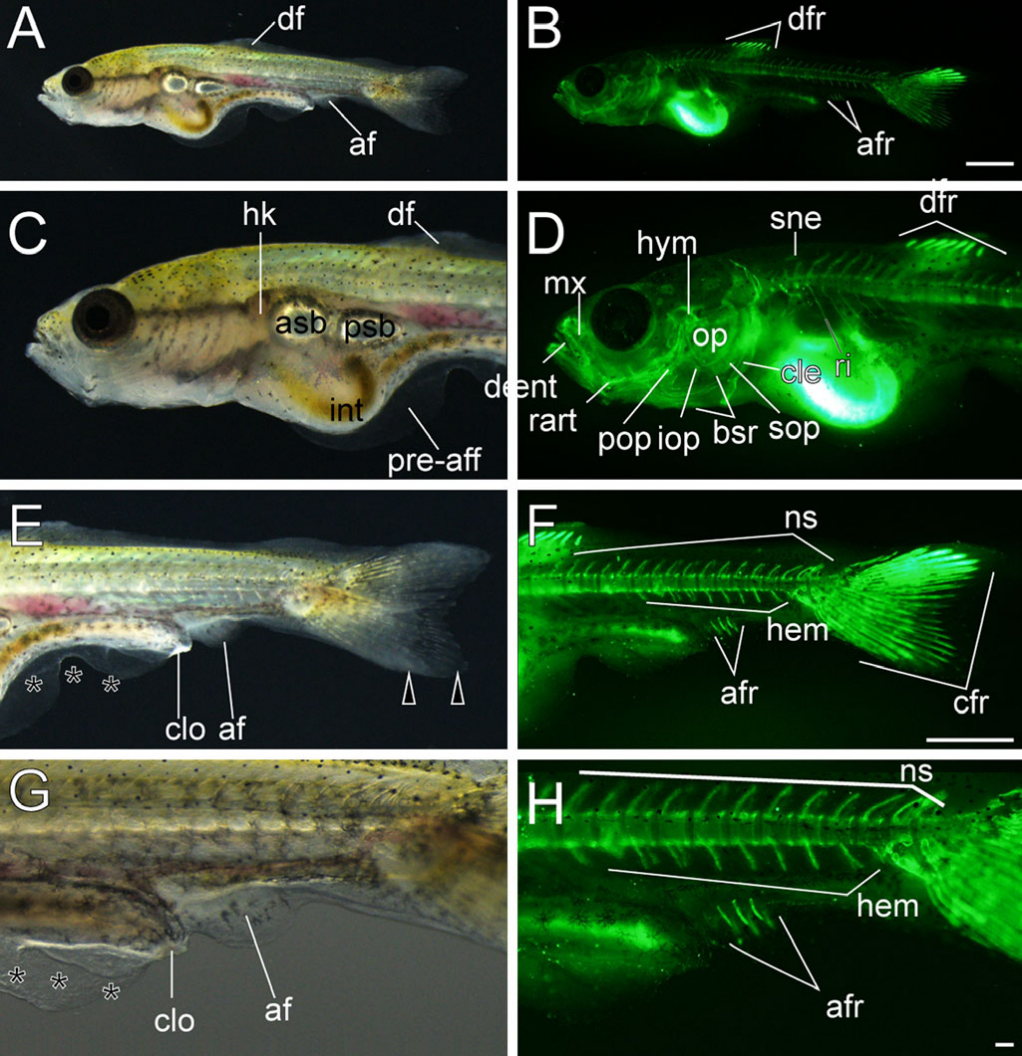
Anal fin ray–stage larvae. **A–H:** Lateral views of three anal fin ray–stage larvae derived from *Oranda* strain. The left column (A,C,E,G) and right column (B,D,F,H) show light and calcein‐stained fluorescent microscopic images. Black arrowheads indicate bifurcated caudal fins. Black asterisks mark mutated area of pre‐anal fin fold. af, anal fin; afr, anal fin rays; asb, anterior swim bladder; bsr, branchiostegal rays; cfr, caudal fin rays; cle, cleithrum; clo, cloaca; dent, dentary; df, dorsal fin; dfr, dorsal fin rays; hem, hemal arch; hk, head kidney; hym, hyomandibular; int, intestine; iop, interopercular; mx, maxilla; ns, neural spine; op, opercular; pop, preopercular; pre‐aff, pre‐anal fin fold; psb, posterior swim bladder; rart, retroarticular; ri, rib; sne, supraneuralis; sop, subopercular. Scale bars B,F = 1 mm. Scale bar H = 0.1 mm. Panels at the first row (A,B), second and third rows (C–F), and forth row (G,H) are shown at the same magnification.

**Figure 17:**
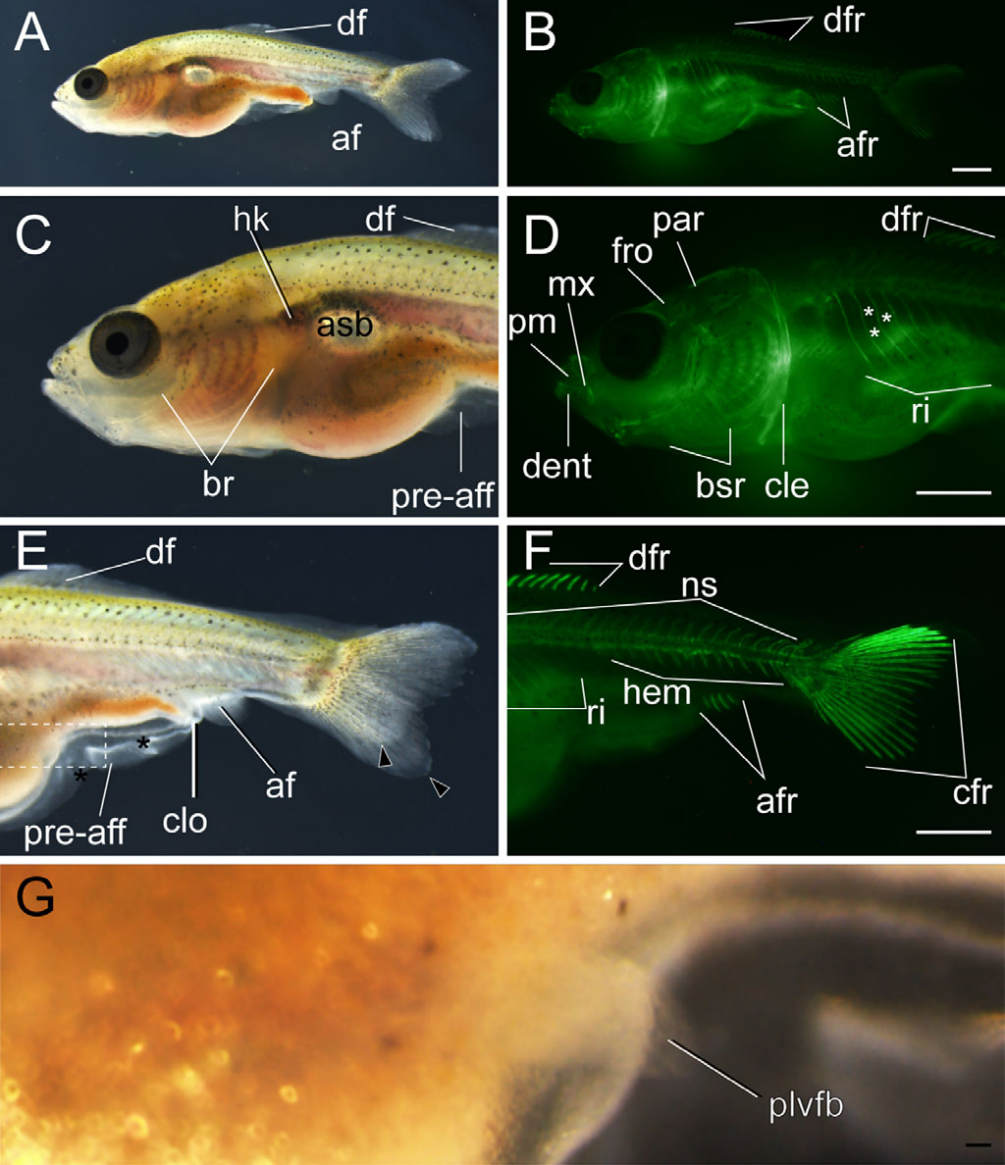
Lateral views of pelvic fin bud–stage larva. **A–B:** Whole‐body view of larva derived from *Ryukin* strain. C–F: Magnified views of anterior (**C,D**) and posterior (**E,F**) regions. **G:** Magnified view of the boxed area in E. Black arrowheads, black asterisks, and white asterisks indicate bifurcated caudal fin, mutated pre‐anal fin fold, and twisted part of ribs, respectively. af, anal fin; afr, anal fin ray; asb, anterior swim bladder; br, branchial; bsr, branchiostegal rays; cfr, caudal fin rays; cle, cleithrum; clo, cloaca; dent, dentary; df, dorsal; dfr, dorsal fin rays; fro, frontal; hem, hemal arch; hk, head kidney; mx, maxilla; ns, neural spine; par, parietal; plvfb, pelvic fin bud; pre‐aff, pre‐anal fin fold; pm, premaxilla; ri, rib. Scale bars B,D,F = 1 mm. Scale bar G = 0.1 mm. Panels at the first row (A,B), second row (C,D), and third row (E,F) are shown at the same magnification.

**Figure 18:**
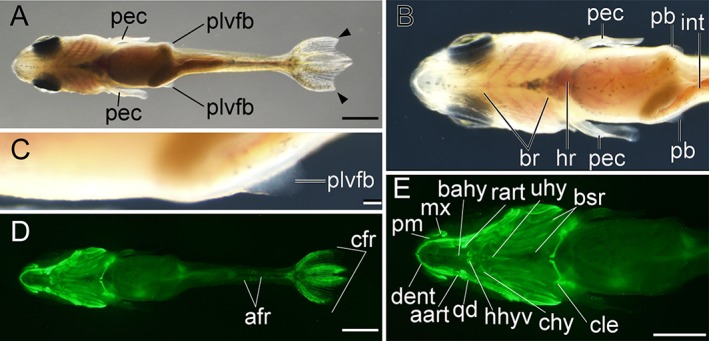
Ventral views of pelvic fin bud–stage larva. **A,D:** Whole‐body views of larva derived from *Ryukin* parents. B,C,E: Magnified views of anterior (**B,E**) and pelvic fin bud (**C**). Bifurcated caudal fin is indicated by black arrowheads. aart, anguloarticular; afr, anal fin ray; bahy, basihyal; br, branchial; bsr, branchiostegal rays; cfr, caudal fin rays; chy, ceratohyal; cle, cleithrum; dent, dentary; hr, heart; hhyv, hyohyal ventral; int, intestine; mx, maxilla; plvfb, pelvic fin bud; pec, pectoral fin; pm, premaxilla; qd, quadrate; rart, retroarticular; uhy, urohyal. Scale bars A,D,E = 1 mm. Scale bar C = 0.1 mm. Panels B,E are shown at the same magnification.

**Figure 19:**
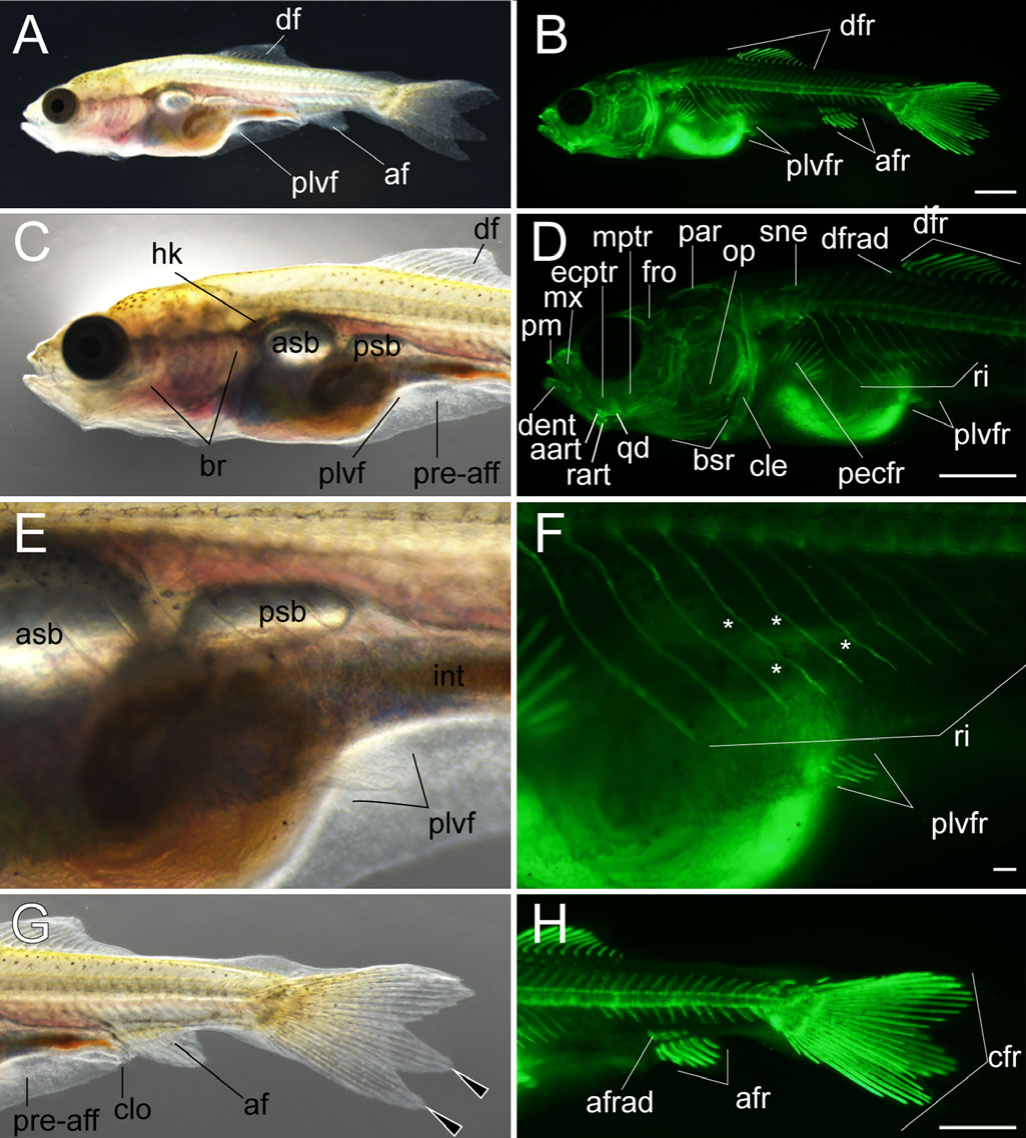
Lateral views of early pelvic fin ray–stage larva. **A,B:** Whole lateral views of larva derived from *Ryukin*‐strain parents. C–H: Magnified views of anterior (**C,D**), pelvic fin (**E,F**), and posterior (**G,H**) regions of A and B. Left column (A,C,E,G) and right column (B,D,F,H) are light and calcein‐stained fluorescent microscopic images. Black arrowheads indicate bifurcated caudal fin. This larva developed four pelvic fin rays. White asterisks mark evidently twisted ribs. aart, anguloarticular; af, anal fin; afr, anal fin rays; afrad, anal fin radials; asb, anterior swim bladder; br, branchial; bsr, branchiostegal rays; cle, cleithrum; clo, cloaca; crf, caudal fin rays; dent, dentary; df, dorsal fin; drf, dorsal fin rays; dfrad, dorsal fin radials; ecptr, ectopterygoid; fro, frontal; hk, head kidney; int, intestine; mptr, metapterygoid; mx, maxilla; op, opercular; par, parietal; pecfr, pectoral fin rays; plvf, pelvic fin; plvfr, pelvic fin ray; pm, premaxilla; pre‐aff, pre‐anal fin fold; psb, posterior swim bladder; qd, quadrate; rart, retroarticular; ri, rib; sne, supraneuralis. Scale bars B,D,H = 1 mm. Scale bar F = 0.1 mm. Panels at the first row (A,B), second row (C,D), third row (E,F), and fourth row (G,H) were photographed at the same magnification.

**Figure 20:**
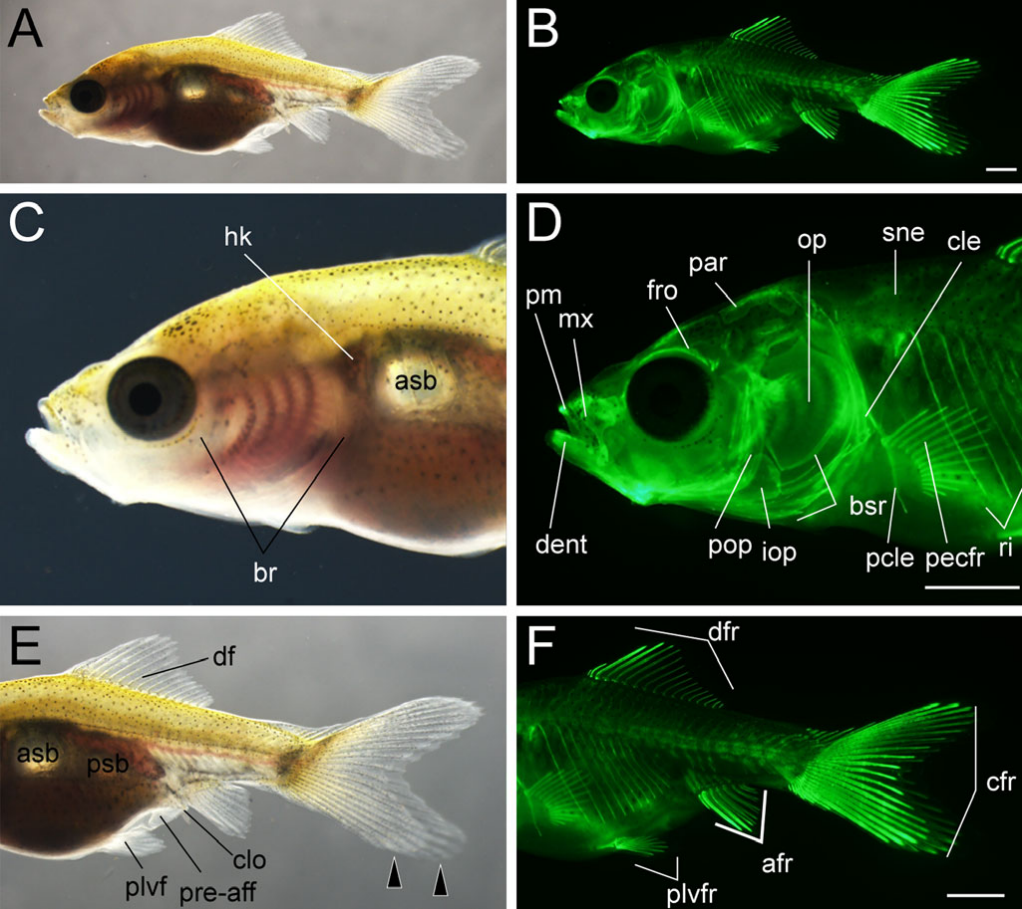
Lateral views of late pelvic fin ray–stage larva. **A,B:** Whole lateral views of larva derived from *Ryukin* parents. C–F: Magnified view of anterior (**C,D**) and posterior (**E,F**) regions of A and B. Left column (A,C,E) and right column (B,D,F) are light and calcein‐stained fluorescent microscopic images. Black arrowheads indicate bifurcated caudal fin. afr, anal fin rays; asb, anterior swim bladder; br, branchial; bsr, branchiostegal rays; cfr, caudal fin rays; cle, cleithrum; clo, cloaca; dent, dentary; df, dorsal fin; dfr, dorsal fin rays; fro frontal; hk, head kidney; iop, interopercular; mx, maxilla; op, opercular; par, parietal; pcle, postcleithrum; pecfr, pectoral fin rays; plvf, pelvic fin; plvfr, pelvic fin rays; pm, premaxilla; pop, preopercular; pre‐aff, pre‐anal fin fold; psb, posterior swim bladder; qd, quadrate; rart, retroarticular; ri, rib; sne, supraneuralis. Scale bars B,D,F = 1 mm. Panels at the first row (A,B), second row (C,D), and third row (E,F) were photographed at the same magnification.

**Figure 21:**
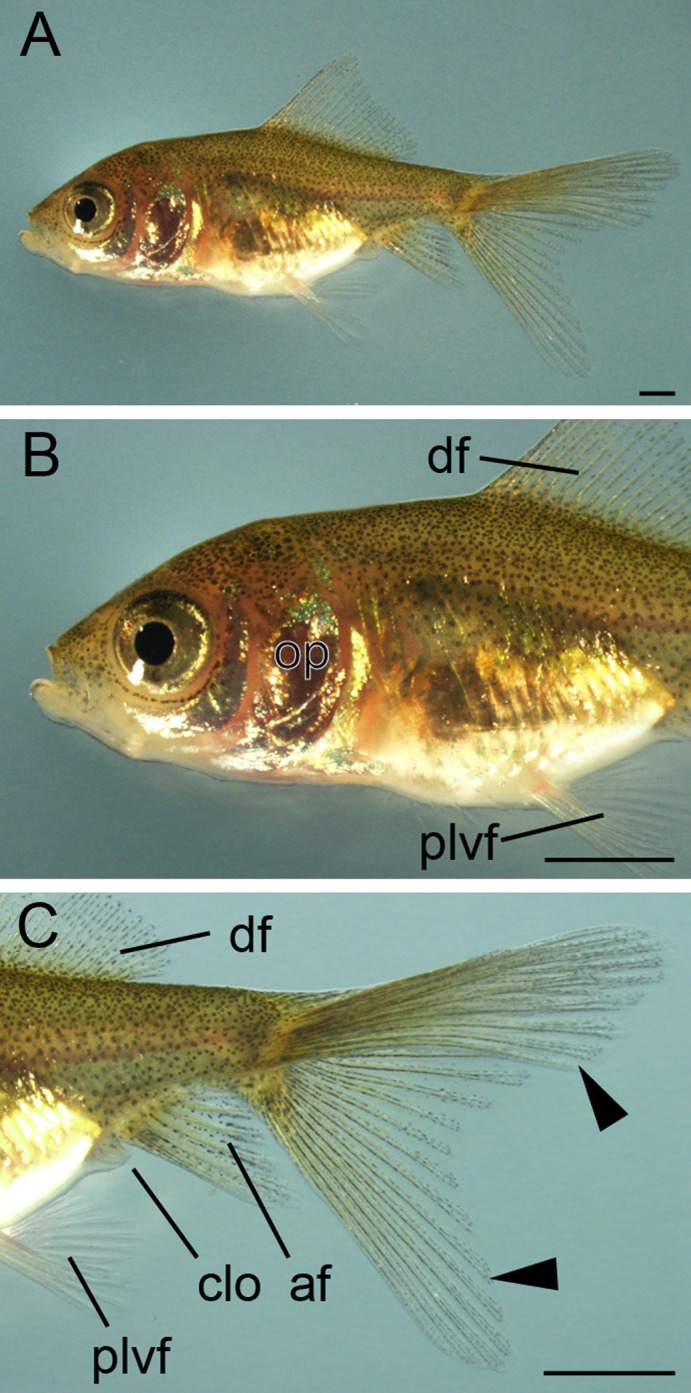
Lateral views of juvenile. **A:** Whole lateral view of juvenile derived from *Oranda* parents. Magnified view of anterior (**B**) and posterior (**C**) regions of A. Black arrowheads indicate bifurcated caudal fin. The entire body is covered by scales. Fin fold is absent at the pre‐cloacal levels. af, anal fin; clo, cloaca; df, dorsal; op, opercular; plvf, pelvic fin. Scale bars = 1 mm.

**Figure 22:**
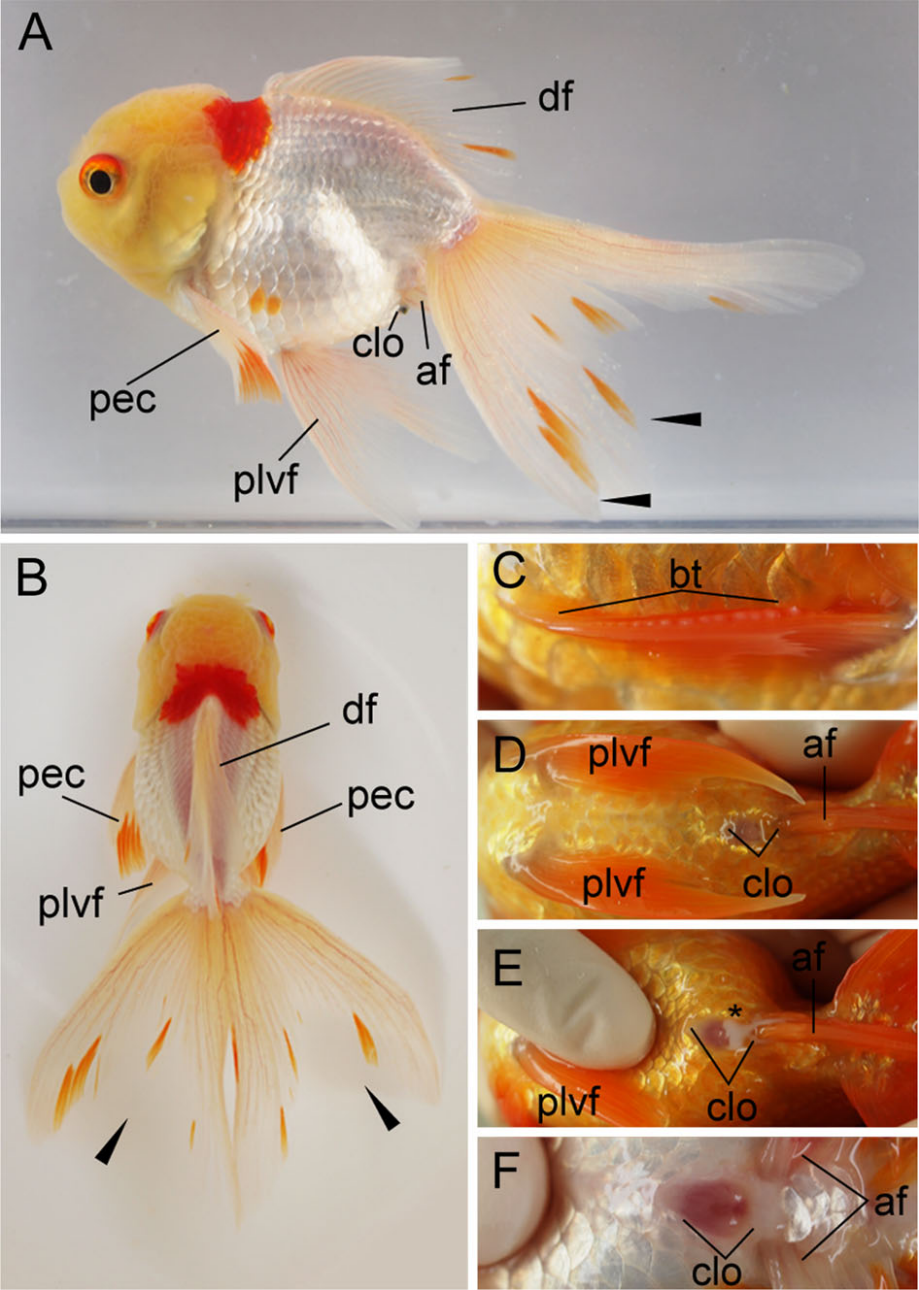
*Oranda*‐strain progenies at 384 dpf. **A:** Lateral view of the female progeny derived from *Oranda* parents. **B:** Dorsal view of the same fish. **C:** Right pectoral fin. Pectoral fins are equipped with breeding tubercles (bt). **D,E:** Ventral views of cloacal region of an adult male specimen. E: Sperm can be observed in the squeezed male, indicated by the black asterisk. **F:** Ventral view of the female fish in panels A,B. The cloaca of the female is more shallow and wider compared to male fish. af, anal fin; bt, breeding tubercles; clo, cloacal; df, dorsal; pec, pectoral fin; plvf, pelvic fin. The pictured male and female fish are approximately 8 cm standard length.

We defined goldfish embryonic and postembryonic stages based on our previous reports describing the normal embryonic staging for single‐tail common goldfish strains (Tsai et al., 2013; Li et al., 2015) (Tables 1, 2). Embryonic stages are categorized into seven periods: zygote, cleavage, blastula, gastrula, segmentation, pharnygular, and hatching (Tsai et al., 2013) (Table 1). Hatching and the presence of protruding‐mouth stages are defined as the embryonic period. The postembryonic stages comprise larval, juvenile, and adult periods (Table 2). The juvenile period is defined by the complete loss of the median fin fold (Li et al., 2015). Based on these previously reported staging indexes, we first described the embryonic and postembryonic developmental process (Figs. 2, 3, 4, 5, 6, 7, 8, 9, 10, 11, 12, 13, 14, 15, 16, 17, 18, 19, 20, 21, 22); the specific features of the twin‐tail goldfish are also summarized in Tables 1 and 2.

**Table 1:** Embryonic Staging Indexes and Twin‐tail Goldfish Specific Features

Period	Representative staging indexes^a^	Specific features of twin‐tail goldfish
Zygote	Perivitelline space, cytoplasm moves to animal pole to form the blastodisc	Under the light microscope, specific features were not detected from zygote to gastrula periods
Cleavage	The number of cells	
Blastula	The shape of the blastodisc	
Gastrula	The shape of the blastoderm	
Segmentation	Somite number, appearance of Kupffer's vesicles, yolk extension, lens and otic vesicles, extended tail and sculpted brain	Enlarged tail bud, polymorphic appearance of Kupffer's vesicles
Pharyngular	OVC, pectoral fin appearance, pigmentation in retina and skin, shape of the median fin fold	Bifurcated median fin folds and enlarged blood island
Hatching	Pectoral fin morphology, xanthophore patterns, caudal fin fold shape	Bifurcated caudal fin fold and expansion of the posterior side of the yolk

a Modified from Tsai et al., 2013

**Table 2:** Postembryonic Staging Indexes and Twin‐tail Goldfish Specific Features

Stage	Representative staging indexes^a^	Specific feature of twin‐tail goldfish
Protruding mouth	Extended mouth, yolk, all fin folds remain; straight notochord at the caudal fin level; heart location moves anteriorly	Bifurcated caudal, anal, and pre‐anal fin folds
Posterior swim bladder	Inflation of the posterior swim bladder; lower jaw extension	Unsegmented calcified tissues at the ventral side of notochord beginning to be visible
Caudal fin ray	Visible caudal fin rays; snout length longer than at Psb; this stage can be divided into substages based on the number of fin rays	Bilaterally bifurcated caudal fin with fin rays; starting to form the globular body shape
Forked caudal fin	Appearance of a largely concave point in the caudal fin, evident anal and dorsal fin condensation; slightly reduced dorsal and post‐anal fin fold	The large concave points in bifurcated caudal fin
Anterior swim bladder	Inflation of anterior swim bladder; enhanced anal and dorsal fin condensation	
Dorsal fin ray	Dorsal fin ray appearance; anterior swim bladder lobe is larger than that in Asb stage	Due to the globular body shape, comparing the sizes of anterior and posterior swim bladders tends to be difficult
Anal fin ray	Anal fin ray appearance; lack of the dorsal fin fold at the anal fin level, anterior swim bladder is larger than posterior swim bladder	Some progenies exhibit bifurcation of anal fin and associated rays
Pelvic fin bud	Pelvic fin bud being visible from lateral side and equipping AER^b^	Bilaterally shifted location of fin bud^c^
Pelvic fin ray	Pelvic fin ray appearance; elongated most posterior dorsal and anal fin rays; trapezoid‐shaped dorsal and anal fins	Globular body shape is more enhanced than the previous stages
Juvenile	Complete loss of the fin fold; posterior serrations at the anterior dorsal and anal fin ray; this stage can be divided into two substages based on completeness of squamation	Strain difference in warty growth beginning to be visible
Adult	Produce mature eggs and sperm	Globular body shape and bifurcated caudal fin

a Modified from Li et al., 2015. ^b^The definition of the pelvic fin bud stage is clarified based on the results of present study (see main text). ^c^The bilaterally shifted location of pectoral fin bud prevents application of this stage to the twin‐tail goldfish progenies (see Fig. 25C,D).

### Embryonic Development: Zygote, Cleavage, Blastula, and Gastrula Periods

Embryonic features were examined in twin‐tail goldfish progenies from zygote to gastrula periods (Figs. 2, 3, 4, 5; Table 1). Under light stereomicroscopy, we could identify each embryonic stage and measure individual developing ratios based on the previously reported staging table (Tsai et al., 2013). From zygote to blastula stages, light stereomicroscopy did not reveal observable twin‐tail goldfish–specific morphologies compared with previously reported single‐tail common goldfish embryonic staging tables (Tsai et al., 2013) (Figs. 2, 3). The twin‐tail goldfish embryos exhibited 512 cells from 3 hr postfertilization (hpf) to 5 hpf (Fig. 3) and reached oblong stages from 5 hpf to 7 hpf (Fig. 3), showing some variation in the developmental rate. Although *Ryukin* embryos derived from two independent clutches were slightly slower to develop from one cell to oblong stages than *Oranda* and the single‐tail common goldfish embryos, we did not detect any specific tendency that would have allowed us to distinguish between twin‐tail goldfish and single‐tail common goldfish between cleavage and blastula stages, based on light stereomicroscopic observations (Fig. 3).

As previously observed in the single‐tail common goldfish (Tsai et al., 2013), the texture of the yolk in cleavage‐ to gastrula‐period embryos was also flexible in the twin‐tail goldfish (Fig. 4). Because of this goldfish‐specific texture of yolk, which is not observed in Zebrafish (Kimmel et al., 1995), the staging index for Zebrafish gastrula‐stage embryos (known as epiboly) was difficult to apply for goldfish embryos; instead, we used the staging index of “blastopore closure” (BC), which has been applied to other teleost species and amphibians (Tsai et al., 2013). In fact, due to the change of the shape of the yolk, the yolk area that was not covered by blastoderm margin was found to change over the course of several seconds (Fig. 4). This flexible yolk of goldfish embryos might explain the high variability in blastopore closure measurements at different hpf (Fig. 5).

Despite the variability, we could detect different tendencies for the *Ryukin*, *Oranda,* and single‐tail common goldfish strains to proceed through the gastrulation process (Fig. 5). The single‐tail common goldfish embryos in this experiment seemed to approximate the previously reported data for single‐tail common goldfish strain of Japan (Tsai et al., 2013). On the other hand, twin‐tail goldfish strains showed differences in developmental timing and ratio of blastopore closure (Fig. 5). *Oranda*‐strain embryos showed some delay in the timing of blastopore closure, although the developmental rate was roughly the same as that of single‐tail goldfish. In contrast to the *Oranda* strain, *Ryukin‐*strain embryos differed from the other strains in both the timing and rate of blastopore closure (Fig. 5). Since all of the embryos were incubated under the same conditions, these results are expected to reflect differences in genetic background (including the *chdA* locus) (Fig. 5).

### Segmentation to Pharyngular Stages

Features specific to twin‐tail goldfish were evident from the segmentation stage onward (Fig. 6A–E; Table 1). At early segmentation stage, the tail bud region of twin‐tail goldfish was enlarged compared to that of single‐tail goldfish (Fig. 6A,C,D). Subsequently, a bifurcated fin fold was observed in the tail bud–equivalent region at the pharyngular stage (Fig. 6C,E,F), consistent with our previous report of expression patterns for dorsal‐ventral patterning–related genes (Abe et al., 2014). Moreover, we recognized significant differences between single and twin‐tail goldfish in the development of Kupffer's vesicle (Fig. 6B–D). Although Kupffer's vesicle was recognized in the twin‐tail goldfish at the 10‐somite stage, it was not detected at later stages (17‐ to 19‐somite stages) under stereomicroscopic observation (Fig. 6B–D). Considering that a reduction in the size of Kupffer's vesicle was reported in the *chordin*‐mutant Medaka (Takashima et al., 2007), it is expected that Kupffer's vesicle in twin‐tail goldfish also exhibits reduced size.

The rate of somite segmentation was linear for all strains (Fig. 7), unlike the varied gastrulation process (Fig. 5). *Oranda‐* and *Ryukin*‐strain embryos showed 2.5‐hr and 4‐hr delay, respectively (Fig. 7). However, the rate was constant among the three strains, with two somites appearing per hour (Tsai et al., 2013), suggesting that the molecular developmental mechanisms of somite segmentation are conserved among the three strains (Jiang et al., 2000; Nikaido et al., 2002; Holley, 2006) (Fig. 7). Pharyngular‐stage embryos showed evident bifurcated caudal fin fold and enlarged blood island, as we previously reported and as others have reported for *chordin*‐mutant Zebrafish (Hammerschmidt et al., 1996; Takashima et al., 2007), suggesting that this tendency is common in *chd*‐depleted teleost species (Table 1). At this stage, the pectoral fin bud can be observed from a lateral view (Fig. 6F). Moreover, in comparison with the single‐tail common goldfish, twin‐tail goldfish embryos exhibit a slightly enlarged yolk extension (Fig. 6F).

### Hatching‐stage Embryos

As we previously reported (Abe et al., 2014), hatching‐stage embryos also showed bifurcated caudal fin fold and expansion of the posterior side of the yolk (Fig. 8; Table 1). Although the caudal fin fold and pre‐anal fin fold exhibited obvious mutant phenotypes, we could not detect any significant phenotypic differences in cranial sensory organs or in pectoral fins of the twin‐tail goldfish we observed (Fig. 8). The pre‐anal fin folds tended to show disrupted morphology at this stage (black asterisks in Fig. 8A–C), and edema was noted in the caudal portion of several progenies (white arrow in Fig. 8C). From the ventral view, we observed the convergence of bifurcated fin folds near the end of the yolk (white arrowheads in Fig. 8D).

Like the single‐tail common goldfish (Li et al., 2015), twin‐tail goldfish embryos hatch at 3–4 days postfertilization (dpf). Thus, we recognize the hatched‐out protruding‐mouth stage as the simultaneous end of embryonic stages and beginning of larval stages, as previously described in the staging table for single‐tail common goldfish (Li et al., 2015) (Figs. 8C,D, 9).

### Developmental Rate of Postembryonic Stages

**Figure 23:**
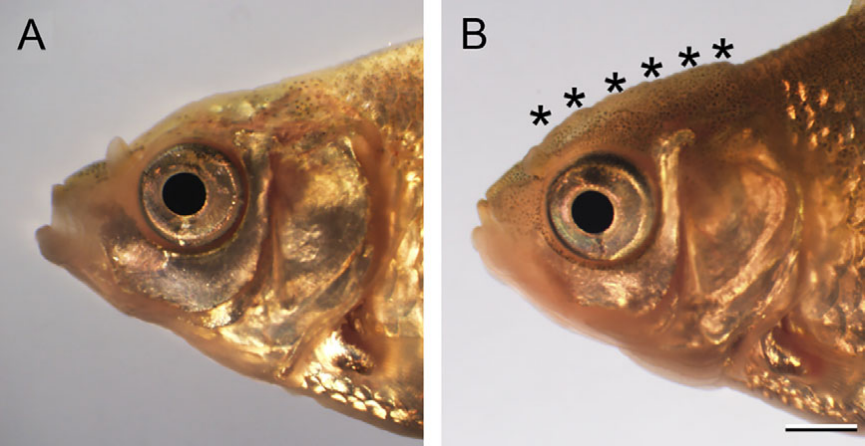
Comparison of cranial regions of *Ryukin* and *Oranda* goldfish progenies at juvenile stage. The lateral view of cranial regions of *Ryukin* (**A**) and *Oranda* (**B**) strain. Black asterisks indicate the warty growth. Scale bar B = 1 mm. Both panels are the same magnification.

**Figure 24:**
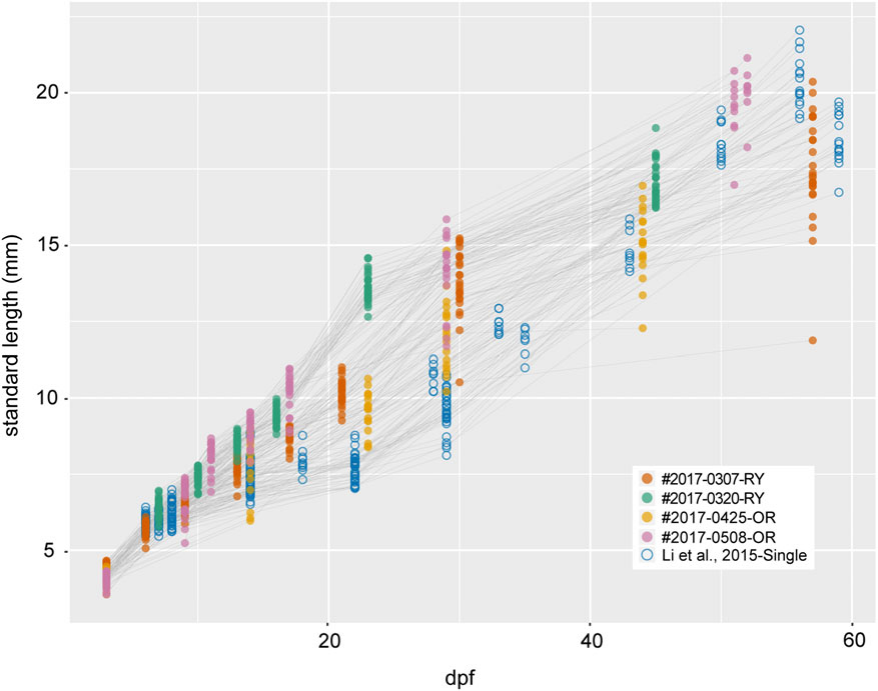
Relationship between standard length and days postfertilization. Points derived from two Ryukin (#2017‐0307‐RY and #2017‐0320‐RY), two Oranda (#2017‐0425‐OR and #2017‐0508‐OR), and the single‐tail common goldfish (Li et al., 2015) are indicated by different colors. A total of 216, 173, 102, 143, and 305 values were derived from 30 #2017‐0307‐RY, 30 2017‐0320‐RY, 27 #2017‐0425‐OR, 28 #2017‐0508‐OR, and 78 the single‐tail common goldfish, respectively.

Based on the single‐tail goldfish staging table (Li et al., 2015), we categorized hatched larvae into protruding‐mouth (Prot), posterior swim bladder (Psb), caudal fin ray (Cr), forked caudal fin (Fcf), anterior swim bladder (Asb), dorsal fin ray (Dr), anal fin ray (Ar), pelvic fin bud (Pb), and pelvic fin ray (Pr) stages; the details of these larval stages are described in Figures 9, 10, 11, 12, 13, 14, 15, 16, 17, 18, 19, 20, 21, 22, 23. Since the development of postembryonic stage progenies is influenced by feeding and maintenance conditions, the relationships between dpf and stages may be variable among progenies, as reported in Zebrafish postembryonic development (Parichy et al., 2009) (Fig. 24).

**Figure 25:**
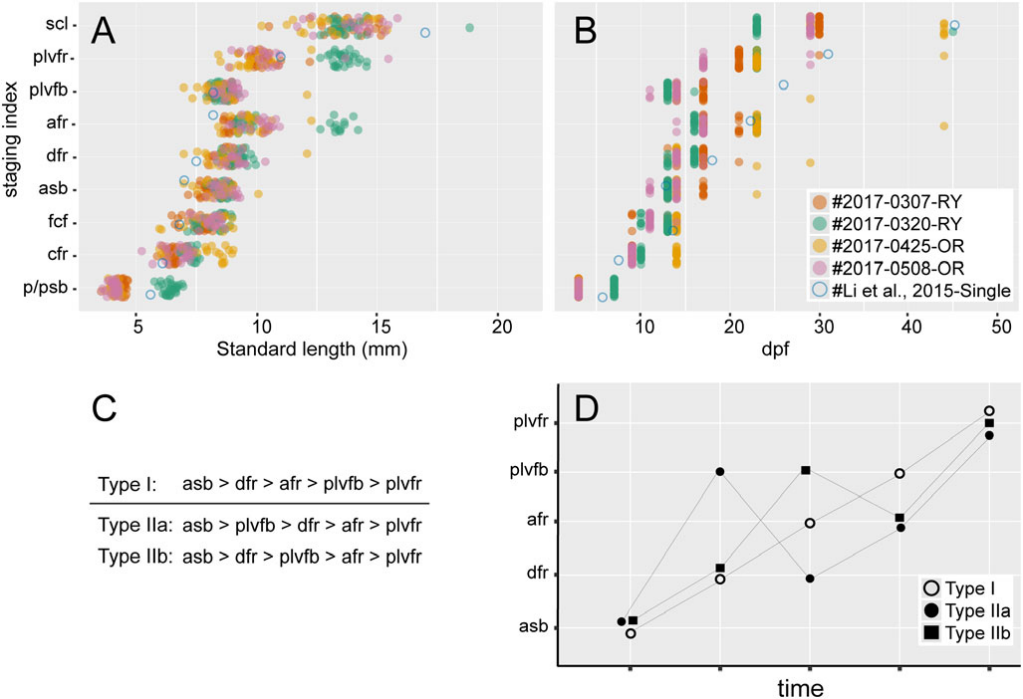
Representative appearance sequence of developmental indexes. **A:** Relationship between standard length and observed staging index. **B:** Relationship between dpf and observed staging index. Appearance sequences of each progeny are represented by different color points. The color‐filled points (3150 points in total) were derived from 30 #2017‐0307‐RY, 30 2017‐0320‐RY, 27 #2017‐0425‐OR, and 28 #2017‐0508‐OR progenies, respectively. The points of the single‐tail common goldfish (blue empty circles) are derived from the postembryonic staging tables (Li et al., 2015). **C:** Two different types of appearance order. **D:** Schematic representation of the relationship between time course and staging index. Type I and Type II larvae were distinguished by the appearance of pelvic fin bud after/before detection of anal fin ray. Type II were further categorized into two subtypes (Type IIa and Type IIb) based on the appearance timing of dorsal fin ray. afr, anal fin fay; asb, anterior swim bladder; cfr, caudal fin rays; dfr, dorsal fin ray; fcf, forked fin robes; plvfb, pelvic fin bud; plvfr, pelvic fin ray; p/psb, protruding mouth or posterior swim bladder; scl, scales cover entire body.

We also evaluated the timing of appearance for features of staging indexes, used for identification of postembryonic single‐tail common goldfish, in progenies from *Oranda* and *Ryukin* parents (Li et al., 2015) (Fig. 25A,B). Although the appearance sequences were almost consistent with those of the single‐tail common goldfish from our previous report (Li et al., 2015), our present observations suggested that the appearance timing of the pelvic fin bud is polymorphic (Fig. 25C,D; see Note on the Timing of Pelvic Fin Bud Appearance).

#### *Protruding‐mouth (Prot) stage*

Beginning at the Prot stage, calcified skeletal elements can be observed under fluorescence microscopy in the cranial area (Fig. 9B–D). The cleithrum and opercular were clearly observed from the lateral aspect, consistent with the skeletal development of single‐tail goldfish (Fig. 9B,D). All of our observed larvae (n = 85) exhibit Prot‐stage phenotype at 3 dpf (approximately 4.2 mm in the standard length).

#### *Posterior swim bladder (Psb) stage*

The posterior swim bladder can be observed from 6 to 7 dpf (6.0 mm in the standard length, approximately) (Fig. 25A,B). At the Psb stage, yolk was reduced in comparison with previous stages (Fig. 10), and some other cranial skeletal elements (including maxilla, anteroarticular, branchiostegal, and ceratohyals) and vertebral elements were recognized (Fig. 10).

#### *Caudal fin ray (Cr) stage*

At the early Cr stage (more than 5 mm standard length), the yolk was completely consumed and the transparency of the body was diminished in comparison with previous stages (Figs. 11, 25A). From this stage, the body began to exhibit a roundish shape, similar to the adult (Fig. 1B,D; Table 2). Most of larvae actively fed on paramecium and brine shrimp; undigested brine shrimp eggs were seen in some larvae (Fig. 11A,C,E). Calcified cranial skeletal elements and vertebral elements were both increased (Fig. 11B,D,F,H,J,L). The pre‐anal fin fold was disrupted or bifurcated (Fig. 11E,M). From the ventral view, bifurcated caudal fin rays could be recognized (Fig. 11N). Furthermore, calcified anterior vertebral elements exhibited clear segmental patterns (Fig. 11F), while in the mid‐trunk the vertebrae were connected by calcified tissues on the ventral side of the notochord (white asterisks in Fig. 11F). In caudal regions, the ventral side of the notochord was calcified (Fig. 11H). From the ventral aspect, calcified cranial skeletons, as well as bilaterally arranged caudal fin rays, could be observed (Fig. 11J,N). Although postcranial skeletons of ornamental twin‐tail goldfish were obviously different from those of single‐tail common goldfish, no significant changes were observed in the cranial skeleton of twin‐tail goldfish (Fig. 11B,D,F,H,J,L,N).

Late‐Cr‐stage larvae were similar to early‐Cr‐stage larvae in terms of body outline, but visible skeletal elements were more apparent in comparison with the previous stage (Figs. 11, 12). Especially, an increased number of visible calcified cranial skeletal elements in late‐Cr‐stage larvae suggested that the calcification of a number of cranial structures occurred in the early to late caudal fin ray stages (Fig. 12B,D). In the trunk, calcified vertebral elements were also increased (Fig. 12B,D,F). In the caudal region, where caudal fin rays appeared, a calcified vertebral element was observed at the level of the flexed notochord (white arrow in Fig. 12F). From the ventral aspect, calcified elongated branchiostegal rays on the cranium, as well as bilaterally duplicated fin fold and caudal fin rays, were clearly detected (Fig. 12G–L).

#### *Forked caudal fin (Fcf) stage*

At approximately 7.5 mm standard length, the twin‐tail goldfish larvae also exhibited a concave point on the caudal fin (Figs. 13, 25A). The concave point, which divides the upper and lower caudal fin lobes, could be observed in both the left and right caudal fins (Fig. 13E,G,K; Table 2). In the fluorescence images, vertebral elements were observed throughout the entire trunk and almost all showed neural spines (Fig. 13D,F). Moreover, ribs and hemal arches appeared in the anterior and posterior trunks, respectively (Fig. 13D,F). The vertebral elements exhibited segmented patterns (Fig. 13D,F), and the number of cranial skeletal elements was increased at this stage (Fig. 13D,F,J). In the observed larva, the caudal fin ray number was quite symmetric; approximately 15 caudal fin rays were observed from a ventral view in both left‐ and right‐side fin lobes (Fig. 13H,L). In addition, anal fin primordia started to appear at this stage (“af” in Fig. 13E).

#### *Anterior swim bladder (Asb) stage*

In goldfish with forked caudal fins, the anterior swim bladder was clearly recognizable (Fig. 14A,C,E). The dual appearance of a concave point in the caudal fin and anterior swim bladder in larvae were found to be closely related to a standard length of approximately 8 mm (Fig. 25A). While the anterior swim bladder was found in larvae with well forked caudal fins, none of the larvae without forked caudal fins had an anterior swim bladder in our investigation (compare Figs. 13C,E, 14C,E,G). This evidence suggests that the appearance order of forked fin fold and anterior swim bladder is the same between twin‐tail goldfish and single‐tail common goldfish (Li et al., 2015). The opaque and prominent region of the dorsal fin fold appears to be the dorsal fin ray developing region (Figs. 14E,G, 15). The intestine showed a curved shape at this stage, although it was quite straight at previous stages (Fig. 14E). The number of ribs and hemal arches were increased (Fig. 14B,D,F,H). Calcified tissues were observed on the dorsal side of the cranial region (Fig. 14D), and although the low fluorescent signals did not allow for precise identification, several cranial bones, including parietal and frontal plates, seem to have begun calcification (Fig. 14D).

#### *Dorsal fin ray (Dr) stage*

Those larvae with anterior swim bladders tended to exhibit dorsal fin rays at 9 mm standard length approximately (Fig. 15A,B, 25A). As such, the photographed larvae exhibited calcified dorsal fin rays between the eleventh and fourteenth vertebrae (Fig. 15C–H). The calcification of cranial and postcranial skeleton progressed in comparison with the previously noted swim bladder–containing larvae (Figs. 14B,D,F,H, 15B,D,F,H). The dorsal fin fold remained in close proximity to the cloacal and anal fin fold (Fig. 15A,E,G). The anterior swim bladder was enlarged compared to the previous stage (Figs. 14C,E, 15C,E).

#### *Anal fin ray (Ar) stage*

Our individual tracing analysis indicated that 7.5‐mm standard‐length larvae tended to exhibit the anal fin rays (Figs. 16A,B,E–H, 25A). Larvae exhibited three pairs of anal fin rays at the 22nd or 23rd vertebrae (Fig. 16A,B,E–H). Simultaneously, the residual dorsal and anal fin folds were smaller, especially the dorsal fin fold proximal to the anal fin (Fig. 16A,E). In the anterior part of the dorsal side, supraneuralis was observed (Fig. 16C,D).

#### *Pelvic fin bud (Pb) stage*

The pelvic fin bud was most often found in larvae that had a multiple number of anal fin rays, as shown in Figures 17 and 18. Although the standard lengths of the pelvic fin bud–positive larvae were also varied (Fig. 25A), approximately, the 8.4‐mm standard‐length larvae tend to exhibit pelvic fin bud (Fig. 17). But smaller larvae also equip pelvic fin bud as shown in Figure 18 (the 8.0‐mm standard‐length larvae).

From a lateral view, the pelvic fin bud was hard to recognize at low magnification (Fig. 17A,C,E,), and even at high magnification the pelvic fin bud was hard to detect due to its transparency (Fig. 17G). On the other hand, from the ventral aspect, the pelvic fin bud was easily recognized (Fig. 18A–C). With a dark background, the pelvic fin bud can be recognized as a pair of opaque membranes at the lateral surface of the trunk (Fig. 18B,C). Our fluorescence imaging revealed a well developed cranial skeleton in the dorsal region (e.g., parietal and frontal plates) and the ventral region (e.g., branchiostegal rays and hyohyal ventral) in comparison with earlier stages (Figs. 17D, 18D,E), showing consistency with previous postembryonic staging (Li et al., 2015). Moreover, twisted ribs and fused vertebral elements were recognized in the fluorescent view (Fig. 17D,F).

#### *Pelvic fin ray (Pr) stage*

Fin rays appeared in the pelvic fin of larvae at approximately 9‐ to 10‐mm length (Figs. 19, 25A). On the lateral surface of the trunk, the pelvic fin and its rays are easily observed (Fig. 19A–F). Dorsal and anal fin folds in the caudal region were reduced, but they remained at the caudal peduncle (Fig. 19G). Fin rays were observed under light microscopy (Fig. 19E). Centra and their attached ribs, neural spine, and hemal spine were well developed in almost all regions of the trunk. The dorsal cranial skeleton was also more clearly recognized (Fig. 19B,D,F,H). In the larvae we examined, four pelvic fin rays were observed (Fig. 19F), and dorsal and anal fin radials were also clearly recognized (Fig. 19B,D,H).

At the late–pelvic fin ray stage, twin‐tail goldfish larvae exhibited reduced fin fold and had scales on the surface of their bodies, but the pre‐anal fin fold still remained (Fig. 20). The globular body shape is more evident than it was in the previous stages (Fig. 20; Table 2). The dorsal, caudal, anal, and pelvic fins had calcified and elongated fin rays (Fig. 20C,D), and the distribution of calcified scales was similar to that previously reported for the single‐tail common goldfish (Fig. 20D,F); calcified scales first appeared on the lateral side of the body. The intensity of calcein fluorescence was different between anterior and posterior portions of the dorsal and anal fins (Fig. 20C–F).

#### *Juvenile and adult stages*

The process of transitioning from larvae to juveniles is similar between the single and twin‐tail goldfish; during this transition, fin folds are reduced and finally the body of juveniles is covered by scales (Li et al., 2015) (Fig. 21). Our tracing of individual fish suggested that this transition tended to occur in progenies, beginning from approximately 12 mm standard length (Fig. 25A). During the juvenile period, the entire body is covered by pigmented tissues, which contain xanthophore, melanophore, and iridophore. The juvenile fish subsequently develop into adults, as illustrated by the 384‐dpf *Oranda*‐strain progeny shown in Figure 22A,B. By inspecting cloaca, we can distinguish between males and females; once males are identified, sperm can be squeezed from the fish (Fig. 22C–E). A wider cloaca is indicative of females (Fig. 22F). Moreover, the male progeny is equipped with breeding tubercles (“bt” in Fig. 22C).

The warty growth, which is a distinguishing ornamental tissue of the *Oranda* strain that is lacking in the *Ryukin* strain, also appeared during the transition from juvenile to adult stages (Figs. 1B,D, 22A,B, 23). We distinguished between *Ryukin‐* and *Oranda*‐strain juveniles by the thickness of dorsal cranial epithelial tissue (black asterisks in Fig. 23B; Table 2). The appearance of a primordial warty growth at this stage indicated that both twin‐tail goldfish develop cranial epithelial tissues at a similar growth rate, but *Oranda* progeny show higher growth rate of the tissues during the juvenile stage (Fig. 23).

### Disrupted Development of Axial Skeletal System

**Figure 26:**
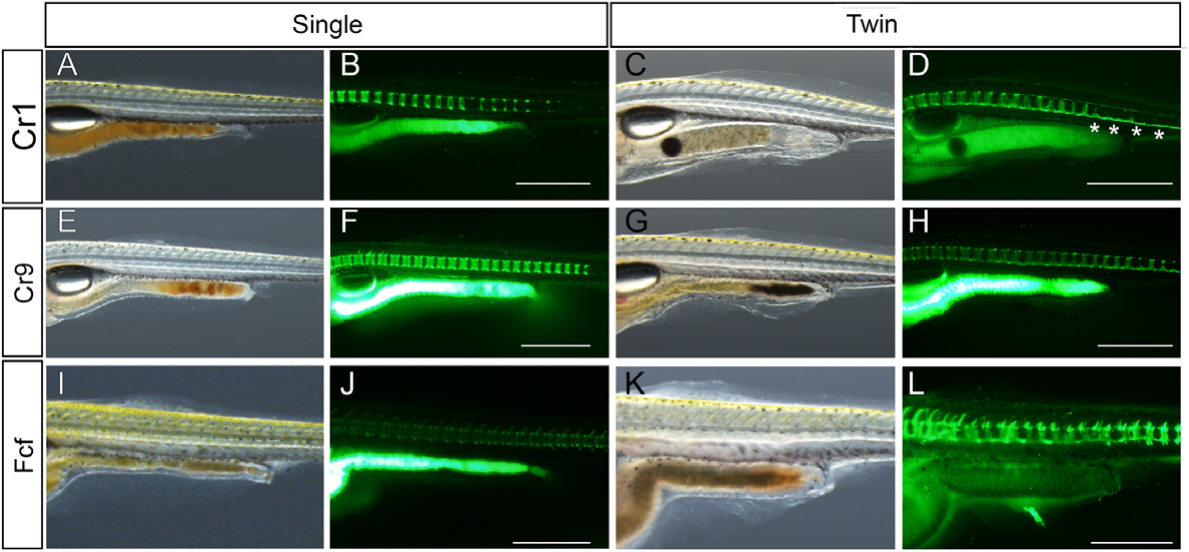
Comparison of vertebral columns of single‐ and twin‐tail goldfish. **A–D:** Early caudal fin ray–stage larvae (Cr1 to Cr2 stages). **E–H:** Nine caudal fin ray–stage larvae. **I–L:** Fcf‐stage larvae. All larvae were photographed from lateral view. Panels C,D show the same larvae as Fig. 11. First and second columns are single‐tail common goldfish progenies. Third and fourth columns are twin‐tail goldfish progenies derived from *Ryukin* parents. Larvae with the same tail type and the same stage are the same larva shown in light and calcein‐stained fluorescence microscopic images. White asterisks in D indicate calcein‐positive notochordal area in which centra are not developed. Scale bars B,D,F,H,J,L = 1 mm. Panels of the same larva [(A,B), (C,D), (E,F), (G,H), (I,J), and (K,L)] were photographed at the same magnifications.

As found in early reports of adult axial skeletal morphology in ornamental goldfish (Koh, 1931, 1932; Asano and Kubo, 1972), most of the ornamental twin‐tail goldfish we examined also exhibited disrupted morphology during development (Figs. 12D,F, 13D,F, 14F,H, 15F,H, 17F, 19D,F). To examine how the process of the axial skeletal formation is different between the single‐tail common goldfish and twin‐tail ornamental goldfish, we compared the goldfish types at early Cr, late Cr, and Fcf stages (Fig. 26). This comparison revealed that each calcified vertebral element was well segmented in the single‐tail goldfish beginning from early Cr to Fcf stages at all axial levels (Fig. 26A,B,E,F,I,J), but the ornamental twin‐tail goldfish larvae showed unsegmented calcein‐positive tissues on the ventral side of the notochord at the Cr stage (caudal fin ray stage shown in Fig. 26C,D,G,H) and disrupted arrangement of vertebral elements (Fig. 26K,L) (Table 2). Based on a report of *dino/chordin* Zebrafish showing similar disruption of the axial skeleton (Fisher and Halpern, 1999), we expect that the disrupted segmentation patterns in ornamental twin‐tail goldfish might be due to the *chdA* ^*E127X*^ allele.

### Histological Observations in Larval Trunk Tissues

**Figure 27:**
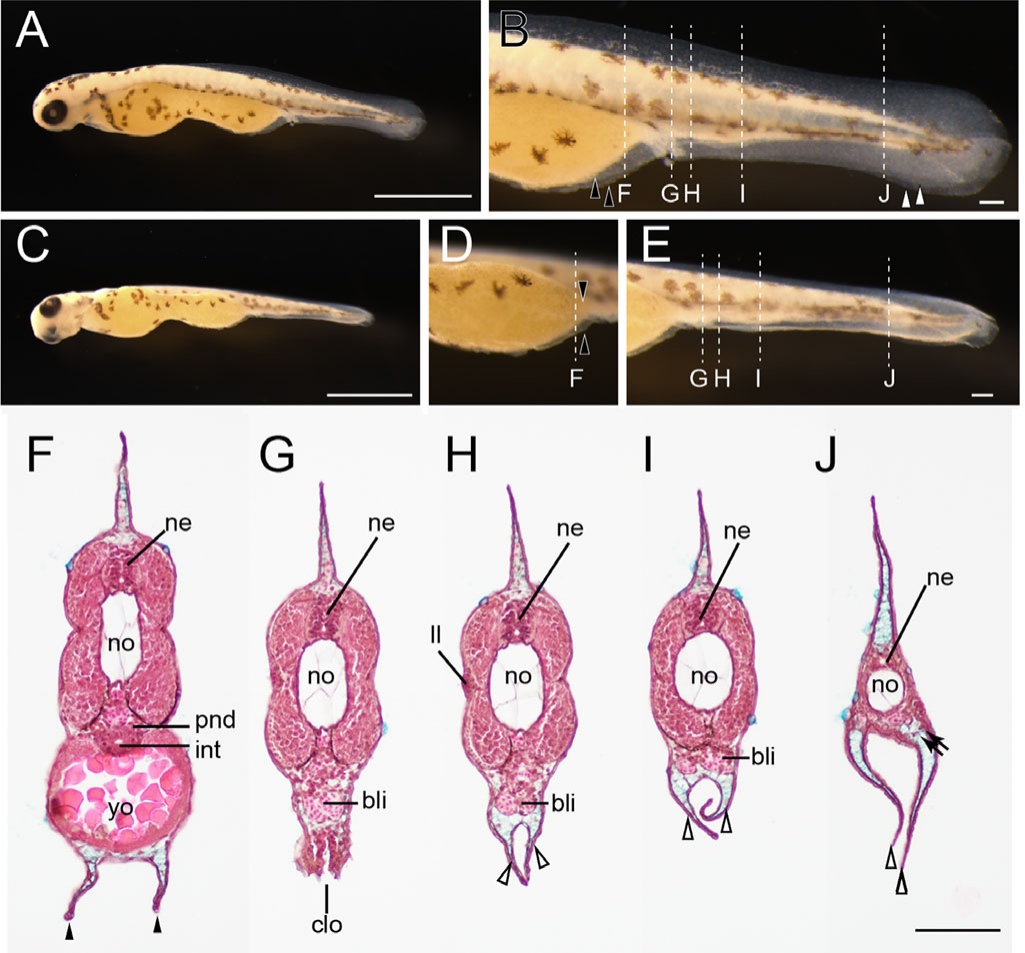
Histological analysis at pre‐/postcloacal levels in a protruding mouth‐stage twin‐tail goldfish larva. **A,B:** Lateral views of protruding mouth‐stage specimen from *Oranda* parents. **C–E:** Lateral oblique views of the same larva as in panel A. F–J: Hematoxylin, eosin, and Alcian Blue–stained transverse sections. **F:** Precloacal region. **G:** Cloacal region. **H–J:** Postcloacal region. Locations of histological sections are indicated by white dashed lines in panels B, D and E. Black arrowheads, white arrowheads, and black arrows indicate bifurcated pre‐anal fin fold, post‐anal fin fold, and migratory mesenchymal cells in the caudal fin, respectively. bli, blood island; clo, cloacal; int, intestine; ll, lateral line; pnd, pronephric duct; ne, neural tube; no, notochord; yo, yolk. Scale bars A,C = 1 mm. Scale bars B,E,J = 0.1 mm. Panels D–J are shown at the same magnifications.

**Figure 28:**
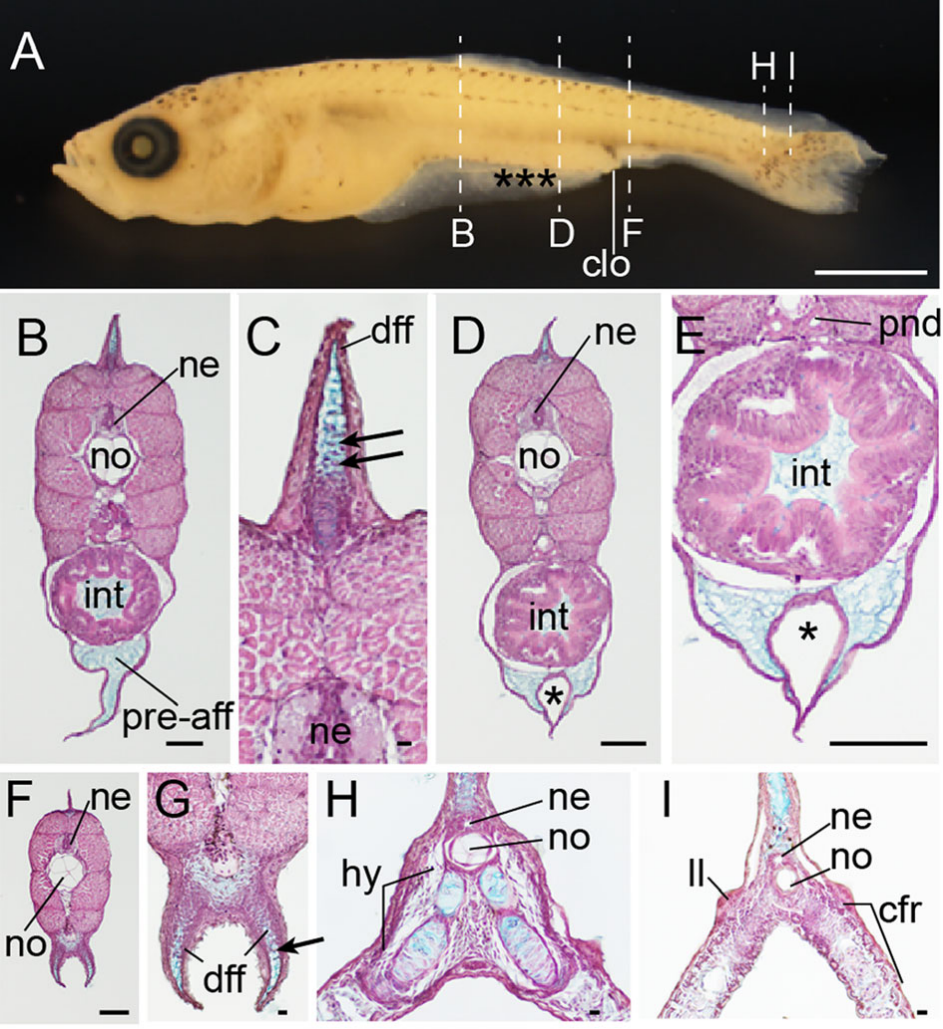
Histological analysis of pre‐/postcloacal levels at Fcf stage. **A:** Whole‐body lateral view of fixed larva from *Ryukin* parents. **B–I:** Hematoxylin eosin and Alcian Blue–stained transverse sections. Panels C,E,G are magnified views of B,D,F, respectively. Locations of each section are marked by dashed white lines in panel A. Black asterisks indicate malformed areas of pre‐anal fin fold. Black arrowheads indicate migratory mesenchymal cells in dorsal and anal fins. cfr, caudal fin rays; clo, cloacal; dff, dorsal fin fold; hy, hypural; int, intestine; ll, lateral line; ne, neural tube; no, notochord; pnd, pronephric duct; pre‐aff, pre‐anal fin fold. Scale bar A = 1 mm. Scale bars B,D,E,F = 0.1 mm. Scale bars C,G,H,I = 0.01 mm.

**Figure 29:**
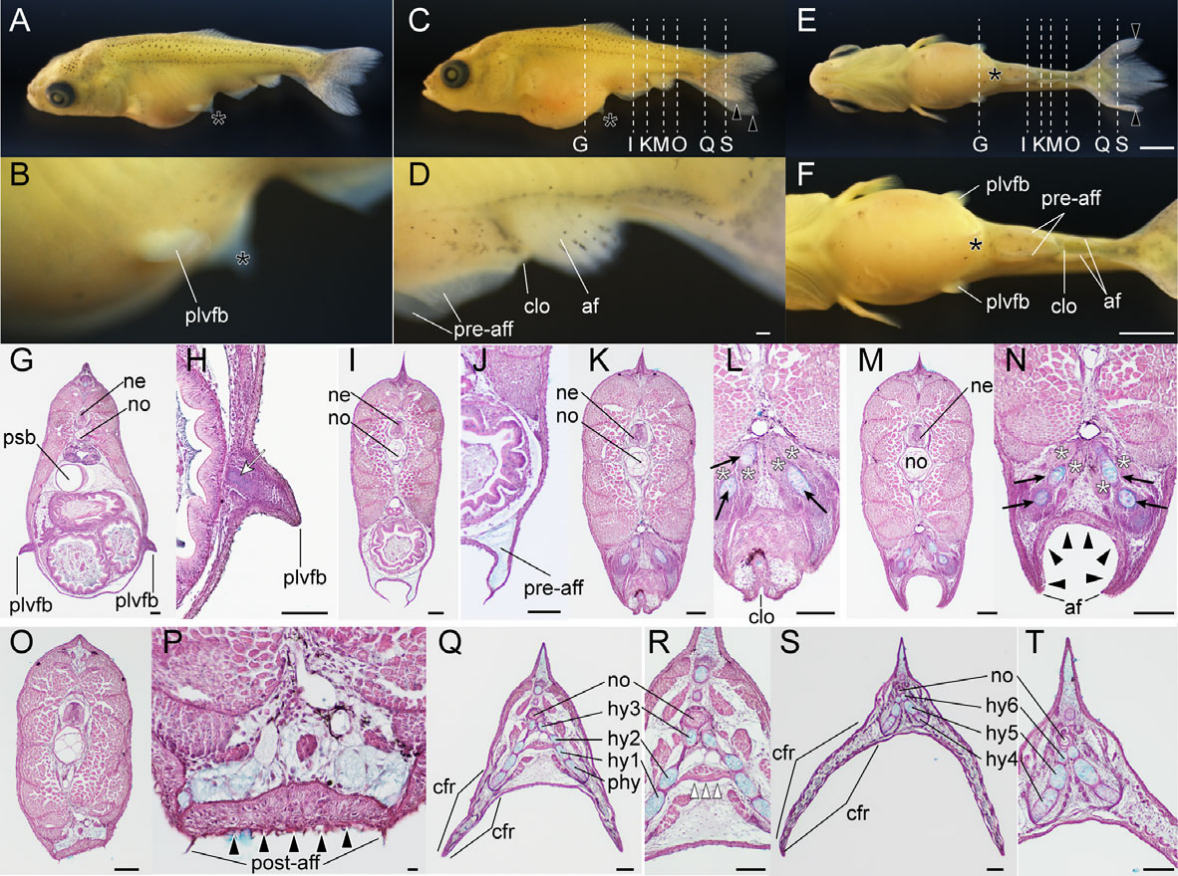
Histological analysis of postcranial regions at pelvic fin bud stage. **A–F:** Fixed larva derived from *Ryukin* parents. **A:** Dorsal oblique view. **C:** Lateral view. **E:** Ventral view. Panels **B,D,F** are magnified views of panels A,C,E, respectively. **G–T:** Hematoxylin and eosin– and Alcian Blue–stained transverse sections. Panels H,J,L,N,P,R,T are magnified views of G,I,K,M,O,Q,S, respectively. Black arrowheads and asterisks indicate thickened epithelial tissue and remaining pre‐anal fin fold in proximity to the pelvic fin bud. Black arrows and white asterisks mark bifurcated skeleton of anal fin (radials) and duplicated muscle attached to skeleton of the anal fin (including depressors and erectors anales). White arrowheads indicate muscular fibers connecting left and right bifurcated second hypurals. White arrow indicates the condensed mesenchymal cells in pelvic fin bud. af, anal fin; cfr, caudal fin rays; clo, cloaca; hy, hypural; ne, neural tube; no, notochord; plvfb, pelvic fin bud; pre‐aff, post‐aff, post anal fin fold; pre‐anal fin fold; psb, posterior swim bladder. Scale bars E,F = 1 mm. Scale bars D,G–T = 0.1 mm. Panels shown the entire larva (A,C,E) and the magnified views (B,D) are shown at the same magnifications.

Our light and fluorescent microscopic analyses revealed that the development of ventral tissues in the caudal region is different between the single‐tail common goldfish and twin‐tail goldfish. To investigate in more detail how the ventral caudal tissues develop in the twin‐tail goldfish, we conducted histological analyses at larval stages (Figs. 27, 28, 29).

In fixed Prot larvae (3.8 mm standard length), we identified dorsal, caudal, and ventral fin folds (pre‐ and post‐anal fin fold) (Fig. 27A,B). In these fin folds, blood vessels and melanocytes were observed (Fig. 27B). At the postcloacal region, an enlarged blood island can be recognized as white tissue (Fig. 27B), and from an oblique view, the bifurcated fin folds at ventral side of the yolk can be observed (Fig. 27C–E). After the location of each tissue was confirmed, the larvae were sectioned along the longitudinal axis, clearly revealing bifurcated pre‐ and post‐anal fin folds and blood cells in the enlarged blood island (Fig. 27F–J). Several mesenchymal cells were also detected in the ventral and caudal fin folds (black arrows in Fig. 27J).

We also observed Fcf‐stage larvae (6.5 mm standard length) (Fig. 28A). These larvae have well developed pre‐anal fin folds, which contain Alcian Blue–positive extracellular matrix but no migratory mesenchymal cells (Fig. 28B–E). On the other hand, dorsal and post‐anal fin folds showed not only Alcian Blue–positive extracellular matrix but also migratory mesenchymal cells (Fig. 28B,C,F,G). In the caudal regions, bifurcated hypural and duplicated caudal fin rays were observed (Fig. 28H,I).

In the further‐developed larvae (9.2 mm standard length), a prominent pelvic fin bud could be clearly observed in the lateral oblique view (Fig. 29A,B). Moreover, remaining pre‐anal fin fold, anal fin, and post‐anal fin fold were recognizable from the lateral view (Fig. 29C,D). The ventral view at the trunk level shows bifurcated features of these tissues (Fig. 29E,F). The pelvic fin buds are located on the lateral side of the trunk region and contain condensed mesenchymal cells (Fig. 29G,H). Bifurcated pre‐anal fin folds also contained Alcian Blue–positive extracellular matrix, but migratory mesenchymal cells could not be detected (Fig. 29I,J). In the cloacal regions, duplicated pterygiophores of anal fin and attached muscle tissue (including depressors and erectors anales) were observed (Fig. 29K–N).

The epithelial tissue at the ventral side of anal fin was thicker than that of the other regions (black arrowheads in Fig. 29M,N). The thick epithelial cells could be recognized in the more posterior regions (black arrowheads in Fig. 29P). Caudal fin skeletons contained Alcian Blue–positive extracellular matrix associated with nonskeletal connective tissues (Fig. 29Q,R). Duplicated muscular elements of caudal fins were also observed, similar to those of anal fins (Fig. 29R). However, the muscular tissue, located in the intermediate region between the left and right second hypural, connected laterally duplicated caudal skeletal elements (white arrowheads in Fig. 29R). In the most posterior sections, calcified fin fold and skeletons were observed (Fig. 29T). The thick epithelial cells, which were observed at the ventral side of the anal fin (Fig. 29S,T), were not observed in caudal regions (Fig. 29Q–T).

### Note on the Timing of Pelvic Fin Bud Appearance

**Figure 30:**
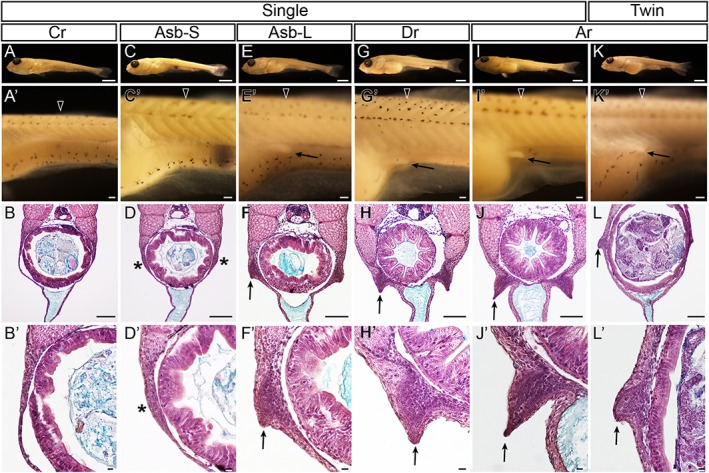
Development and variation of pelvic fin bud in single‐ and twin‐tail goldfish. A–J’ and K–L’ show single‐tail common goldfish and *Ryukin* progenies, respectively. **A,C,E,G,I,K** show oblique lateral views of fixed goldfish larvae. **A’,C’,E’,G’,I’,K’** are magnified views of first‐row panels. **B,D,F,H,J,L** are hematoxylin and eosin– and Alcian Blue–stained sections of larvae in first‐row panels. **B’,D’,F’,H’,J’,L’** show magnified views of left lateral side in third‐row panels. First, second, third, and fourth columns show caudal fin ray–stage larva, small larva at anterior swim bladder stage, large larva at anterior swim bladder stage, and dorsal fin ray–stage larva, respectively. Fifth and sixth columns show anal fin ray–stage larvae. Locations of histological sections are marked by black arrowheads in the second row. Black arrows in panels E’,G’,I’,J,L’ indicate the most posterior extent of pelvic fin buds. Black asterisks indicate pelvic fin bud primordia. Arrows in panels F,F’,H,H’,J,J’,L,L’ indicate apical ectodermal ridges of pelvic fin bud. Scale bars first row = 1 mm. Scale bars second and third rows = 0.1 mm. Scale bars fourth row = 0.01 mm.

In our previous study, we reported that the pelvic fin bud could be detected after the anal fin ray appearance (Li et al., 2015). This observation suggested that the appearance order of anal fin and pelvic fin bud of goldfish is consistent with that of another closely related teleost species, Zebrafish (Parichy et al., 2009). However, our current results from individual tracing in *Oranda* and *Ryukin* progenies showed an inconsistency with the postembryonic staging table of the single‐tail common goldfish (Fig. 25C,D) (Li et al., 2015). In total, 75 of 115 larvae exhibit pelvic fin bud before the appearance of anal fin ray and/or dorsal fin ray (Fig. 25C,D). We categorized larvae into two types (Type I and II) based on the appearance timing of pelvic fin bud (Fig. 25C,D). Although all previously observed single‐tail common goldfish were categorized as Type I, our observed twin‐tail goldfish were mostly categorized as Type II. This inconsistency seems to indicate that there is a difference between the single and twin‐tail goldfish in the sequence of appearance. However, we still cannot distinguish whether the differences are derived from intra‐ or interstrain polymorphisms, or if different experimental conditions between present and previous studies affected the sequence of appearance (Li et al., 2015) (Fig. 25C,D). Thus, we examined the development of the pelvic fin bud in the single‐tail common goldfish in the Cr‐ to Ar‐stage larvae and compared it to that in the twin‐tail ornamental goldfish at the histological level (Fig. 30).

Our histological analysis indicated that the pelvic fin bud appeared before the anal fin ray appearance in the single‐tail common goldfish (Fig. 30A–H’). Evidence of a pelvic fin bud was not observed in histological sections, consistent with the stereomicroscopic observations in the Cr‐stage larvae (Fig. 30A–B’). But the progenies having prominent anterior swim bladder also showed protrusions in the epithelial tissues lateral to the intestine (Fig. 30C–F’). Although the apical ectodermal ridge (AER) was not evident in the larva with small anterior swim bladders (Fig. 30D,D’), this morphological structure was clearly recognized in the larvae with large anterior swim bladders (Fig. 30F,F’). Subsequently, the AER became more evident in larvae with dorsal fin rays, and the pelvic fin buds were located at the lateral ventral body surface (Fig. 30G–H’). Finally, the pelvic fin bud of the single‐tail common goldfish progeny forms a fine and thin AER at later stages (Fig. 30I–J’).

These results imply that the pelvic fin bud can be formed before the formation of dorsal and anal fin rays in the single‐tail common goldfish strain (Fig. 30C–J’), suggesting that both twin‐tail goldfish and single‐tail goldfish can form pelvic fin buds before the formation of anal fin rays (Fig. 25). However, it is still unclear why previously observed ontogenetic sequences of anterior swim bladder, dorsal fin ray, anal fin ray, and pelvic fin bud are not consistent with our current results.

**Figure 31:**
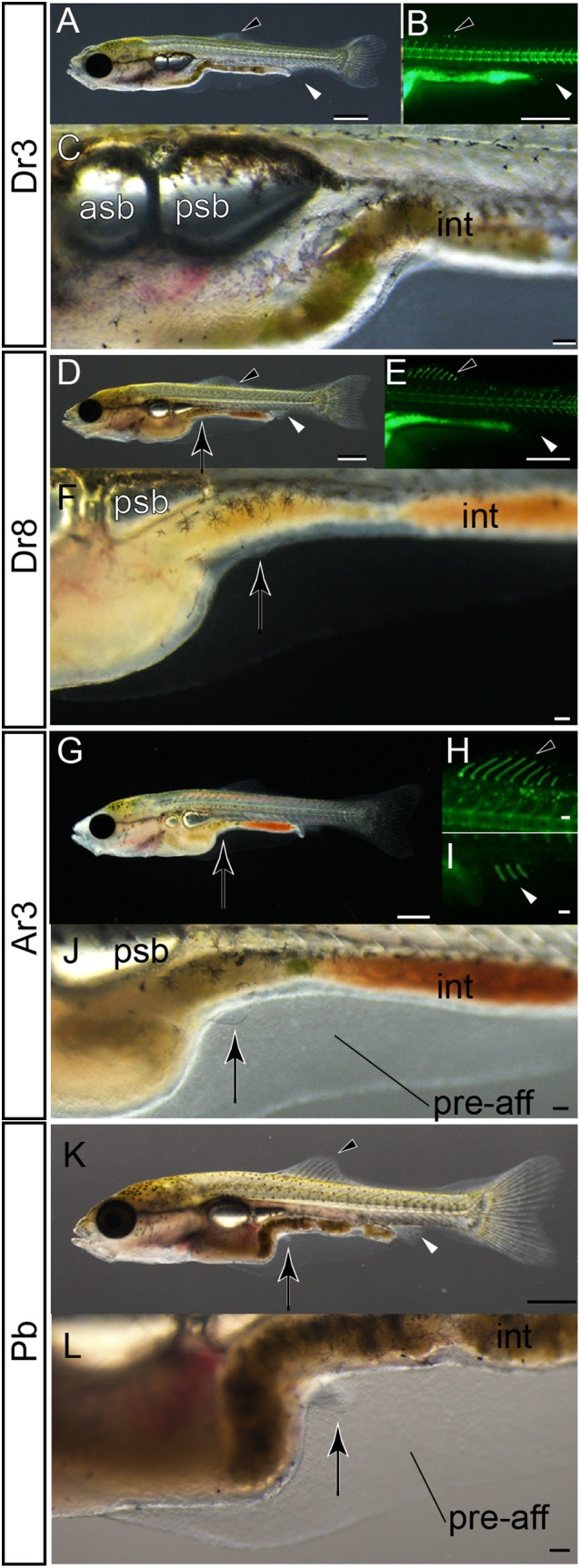
Pelvic fin bud development in single‐tail goldfish. **A–C:** Lateral views of a three dorsal fin ray–stage larva. **D–F:** Lateral views of an eight dorsal fin ray stage–larva. **G–J:** Lateral views of a three anal fin ray–stage larva. **K,L:** Lateral views of a pelvic fin bud–stage larva. Panels B,E,H show fluorescence. Panels C,F,J,L are magnified views of A,D,G,K. Black and white arrowheads indicate dorsal and anal fins, respectively. The larva in panels D–F is the same larva depicted in Fig. 30G,G’,H,H’. Black arrows mark pelvic fin or its primordia. Standard length of larvae in panels A–C is 8.2 mm, D–F is 9.0 mm, G–J is 8.8 mm, and K–L is 8.7 mm. asb, anterior swim bladder; int, intestine; pre‐aff, pre‐anal fin fold; psb, posterior swim bladder. Scale bars A,B,D,E,G,K = 1 mm. Scale bars C,F,H,I,J,L = 0.1 mm.

To address this question, we reexamined the development of single‐tail common goldfish (Fig. 31). In live larvae of 8.2‐mm standard length with well developed anterior swim bladders and three dorsal fin rays (Fig. 31A–C), we could not observe any prominent pelvic fin bud (Fig. 31C), although we did observe pelvic fin buds in fixed Asb‐stage larvae (Fig. 30D,D’). Moreover, in the larger larvae (9.0 mm standard length), which were used for histological sectioning (Fig. 30G–H’), we could not detect prominent pelvic fin buds from the lateral view (Fig. 31D–F). These results implied that the transparency of the pelvic fin bud impeded our ability to detect the subtle protrusion of the pelvic fin bud in intact animals (Fig. 31D–F) even though the protrusion can be recognized at the histological level (Fig. 30H,H’).

This difference between histological and light stereomicroscopic observations was detected in the larvae (8.8 mm standard length), which had already developed anal fin rays (Fig. 31G–J). Although we also observed the pelvic fin bud by histology in larvae with anal fin rays (Fig. 30J’), the pelvic fin bud was difficult to recognize from the lateral view in live fish samples due to its transparency (Fig. 31J). However, in larvae of almost the same size (8.7 mm standard length), easily recognized pelvic fin buds with well developed AER and condensed mesenchymal cells could be observed (Fig. 31K,L). Based on the sizes of these examined larvae, we conclude that the timing of anal fin ray and pelvic fin bud appearance are closely related to each other and the order is readily interchangeable during the ontogenetic process. In other words, these variations in timing of pelvic fin bud appearance represent intraspecies (intra‐/interstrain) polymorphisms, as was previously reported in a cichlid species (*Haplochromis piceatus*) (de Jong et al., 2009).

Moreover, it should be noted that the methodologies of microscopic observation determine the ability to appropriately identify developmental stages. Considering that the presence/absence of the pelvic fin bud was previously distinguished based on photographs taken from the lateral view (Li et al., 2015), we suspect that the identified fin buds were ventrally located pelvic fin buds that tend to be found after the development of the anal fin rays in the single‐tail goldfish (Li et al., 2015). On the other hand, twin‐tail goldfish tend to form pelvic fin buds in a relatively lateral position on the body (Fig. 30K–L’). More specifically, at the equivalent stage, the well developed pelvic fin bud of the single‐tail common goldfish is attached to the pre‐anal fin fold (Fig. 30I–J’), while the pelvic fin bud of twin‐tail goldfish with AER and condensed mesenchyme is located on the lateral surface of the fish body (Fig. 30K–L’).

These differences suggest that the pelvic fin bud of the single‐tail goldfish can be more easily observed from a lateral view than that of the twin‐tail goldfish, which is better detected from oblique lateral, dorsal, and ventral views in the live samples (Figs. 17, 18). Thus, the differences in the location of the pelvic fin bud may potentially create an artificial bias in the identification of the appearance timing of the pelvic fin bud. This issue caused several problems related to the definition of the Pr stage for the staging index. One of the problems is the closely related timing of appearance for the anal fin ray and detection of the pelvic fin bud (Figs. 30, 31). This problem may be resolved by redefining the Pb stage as follows: Pb stage has a pelvic fin bud with AER that is evidently recognized from a lateral view (Fig. 31G–L). This redefinition of the Pb stage allows the continued use of the previously established staging index for single‐tail common goldfish without significant modifications.

However, even after redefining the Pb stage, the artificial bias derived from uncertain detection of the transparent pelvic fin bud is still hard to avoid. This concern is especially relevant when comparing the single‐ and the twin‐tail goldfish strains, since the location of the pelvic fin bud differs between the strains (Figs. 17, 18). Thus, to avoid confusion, it seems better to dismiss the Pr stage for stage identification of twin‐tail goldfish. In addition to these technical problems, the application of the single‐tail common goldfish postembryonic staging table raised other considerable problems related to variations in morphology and ontogenetic sequence (see Discussion).

### Variations in Morphology in Postembryonic Stages

**Figure 32:**
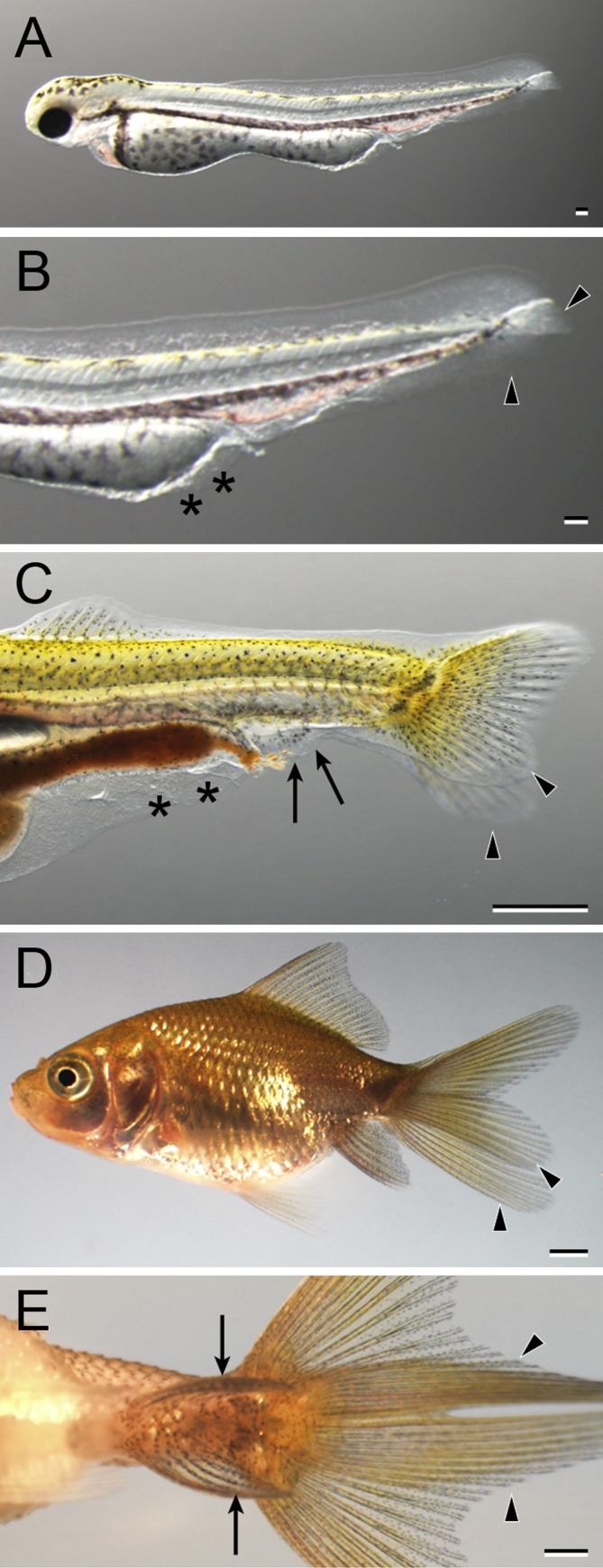
The developmental progression of bifurcated anal fin and caudal fin (2A2C) in *Oranda* progeny. **A:** Whole‐body lateral view of a hatched larva (3 dpf). **B:** Magnified view of the caudal region of the larva in panel A. **C:** Lateral view of the caudal region of Fcf larva (14 dpf). **D:** Whole‐body lateral view of a juvenile (51 dpf). **E:** Ventral view of the caudal region of the juvenile in panel D: Black arrowheads, black arrows, and black asterisks indicate bifurcated caudal fin, bifurcated anal fin, and malformed fin fold of pre‐anal level, respectively. Scale bars A,B = 0.1 mm. Scale bars C,D,E = 1 mm.

**Figure 33:**
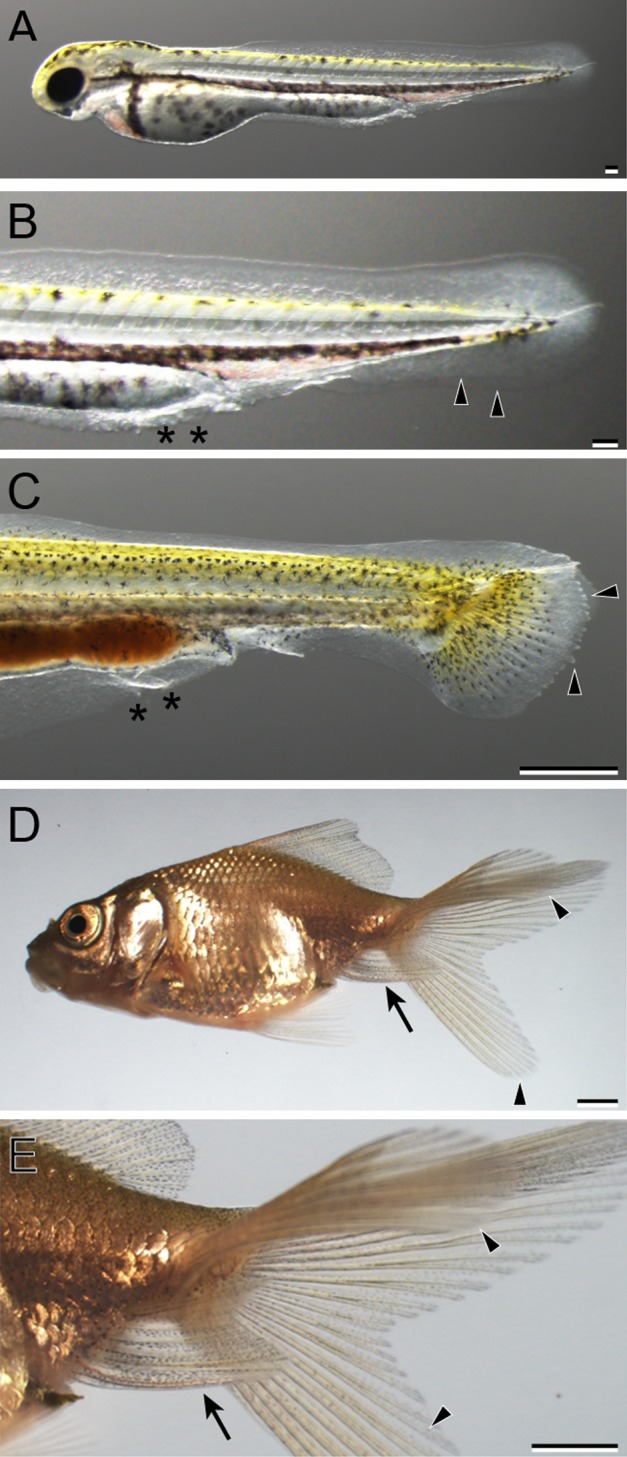
The developmental progression of the single anal fin and bifurcated caudal fin (1A2C) in *Oranda* progeny. **A:** Whole‐body lateral view of a hatched larva (3 dpf). **B:** Magnified view of the caudal region of the hatched larva in panel A. **C:** Lateral view of caudal portion of a Cr‐stage larva (11 dpf). **D:** Whole‐body lateral view of juvenile (51 dpf). **E:** Magnified view of caudal region of the juvenile in panel D: Black arrowheads indicate caudal fin fold and caudal fin. Black arrows and asterisks indicate the anal fin and malformed region of pre‐anal level. Scale bars A,B = 0.1 mm. Scale bars C,D,E = 1 mm.

**Figure 34:**
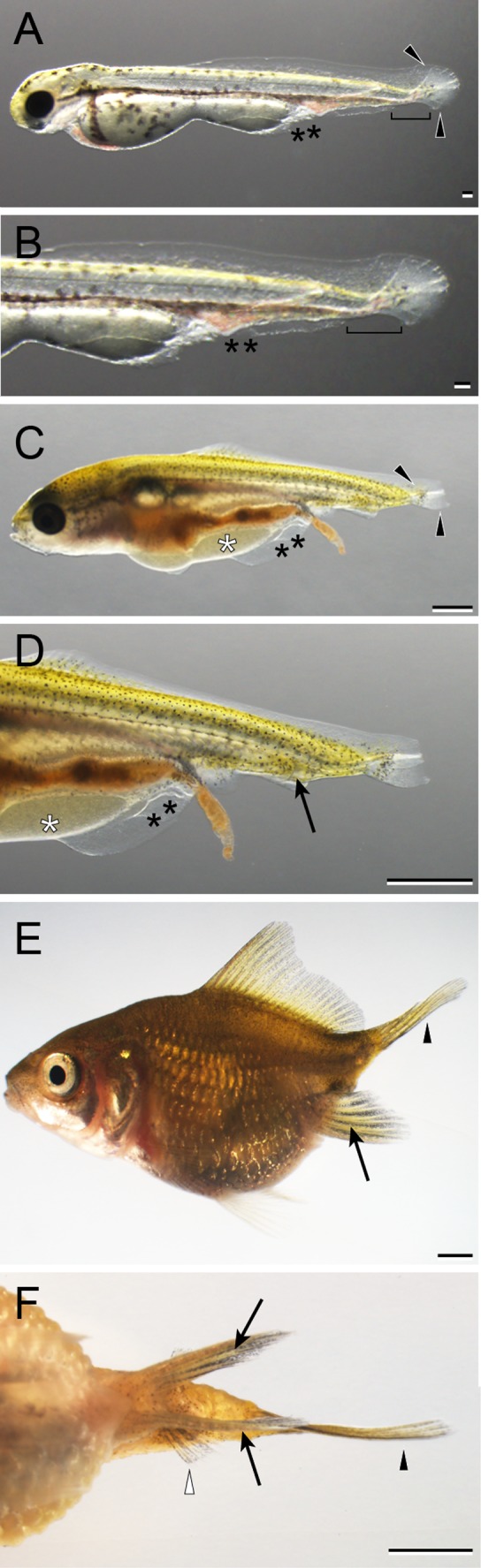
The developmental progression of bifurcated anal fin and reduced caudal fin in *Oranda* progeny. **A:** Whole‐body lateral view of a hatched larva. **B:** Magnified view of the caudal region of the larva in panel A. **C:** Lateral view of Dr stage (14 dpf). **D:** Magnified view of the caudal region of the larva in panel C. **E:** Lateral view of juvenile (51 dpf). **F:** Magnified ventral view of the juvenile in panel E. Black arrowheads indicate caudal fin fold (and caudal fin); black arrows indicate anal fin; black asterisks indicate malformed region of pre‐anal fin fold. White asterisks indicated enlarged ventral region in panels C,D. A missing portion of the post‐anal fin is indicated by the black bracket in panels A,B. A white arrowhead points to the rudimental ventral fin lobe in panel F. Scale bars A,B = 0.1 mm. Scale bars C–F = 1 mm.

**Figure 35:**
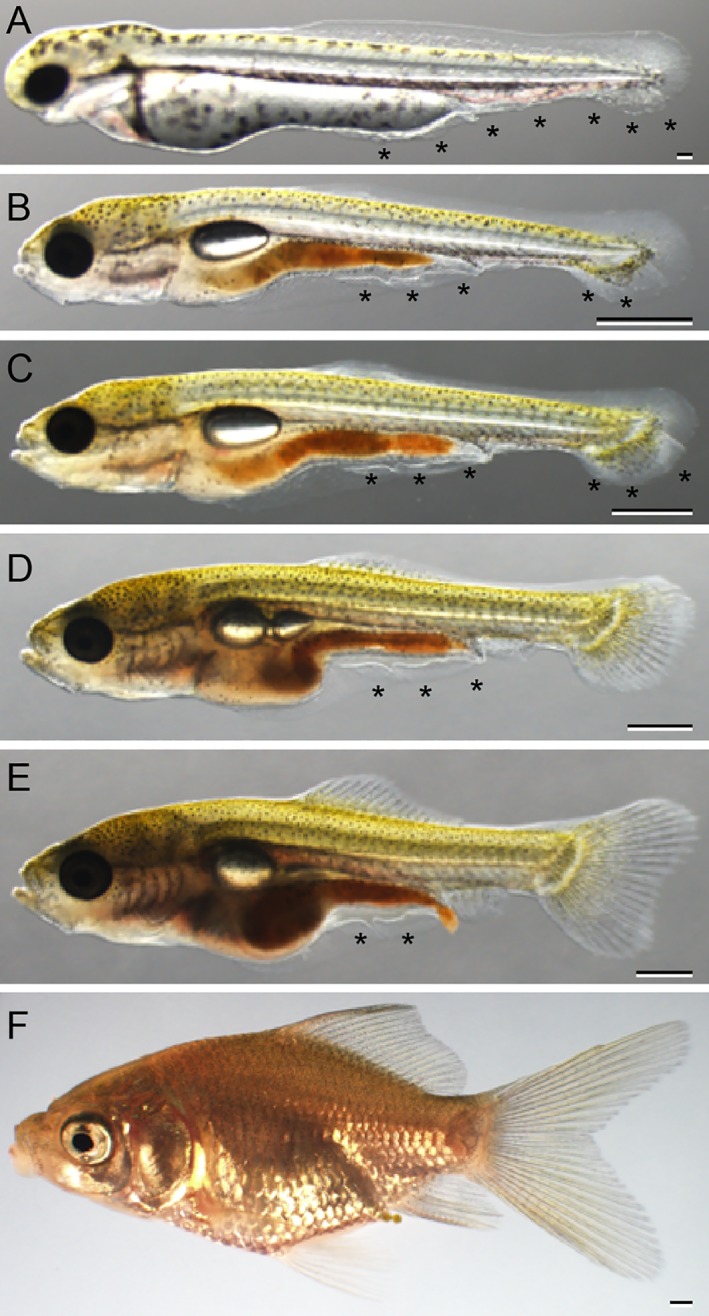
The developmental process of the single caudal fin *Oranda* progeny. **A:** Hatched larva (3 dpf). **B:** Early Cr stage (9 dpf). **C:** Late Cr stage (11 dpf). **D:** Dr stage (14 dpf). **E:** Pb stage (17 dpf). **F:** Juvenile stage (51 dpf). Black asterisks indicate areas with malformed fin fold. Scale bar A = 0.1 mm. Scale bars B–F = 1 mm.

Progenies of ornamental twin‐tail goldfish strains used for the developmental characterization were highly similar to their parents at the late larval and juvenile stages with regard to postcranial morphological features; most of the progenies exhibited bifurcation of the caudal fin (Figs. 1B,D, 19, 20, 21). However, we also noted several variations in anal and caudal fin morphologies and development (Figs. 32, 33, 34, 35). To investigate in detail how these variations arise, we individually traced the development of the different progeny types, showing the development of different types of postcranial morphologies (Figs. 32, 33, 34, 35; Table 3).

**Table 3:** Caudal Fin Morphology of OR and RY Progenies From 41 dpf to 57 dpf

Clutch number	Bifurcated	Single	Reduced	Total
#2017‐0307‐RY	25	0	0	25
#2017‐0320‐RY	29	0	0	29
#2017‐0425‐OR	17	1	1	19
#2017‐0508‐OR	16	1	1	18
Total	87	2	2	91

In total, four clutches, consisting of 91 progenies (Pr to juvenile stage), were used to observe caudal fin morphology (Table 3). Of those, 87 showed bifurcated caudal fin, two had reduced caudal fin robe, and the other two were had a single caudal fin with a postcloacal phenotype; the representative morphologies are shown in Figures 32, 33, 34, 35. We also compared the ratio of bifurcated‐ to single‐caudal‐fin morphology to our present study of twin‐tail goldfish progenies and backcross progenies from a previous study (Table 4). In total, 42 of 338 *chdA* ^*E127X/E127X*^ progenies, derived from the crossing of an ornamental twin‐tail goldfish male and a hybrid female (*chdA* ^*wt/E127X*^), exhibited a single‐caudal‐fin phenotype in our previous research (Abe et al., 2014). This high occurrence was significantly different from our present population of twin‐tail goldfish progenies in the expressivity of bifurcated‐caudal‐fin phenotype (Table 4).

**Table 4:** Comparison Between Twin‐tail and Backcross Populations

Populations	Bifurcated	Single	Total
2017‐Twin^a^	87	2	89
2014‐Backcross^b^	296	42	338
Total	383	44	427
Chi‐square test, *P* < 0.05			

a Two reduced caudal fin progenies were removed from Table 1. ^b^Abe et al., 2014.

For more detailed morphological analysis, we divided the twin‐tail goldfish progenies with bifurcated caudal fin into two different groups based on anal fin morphology; the first group comprised progenies with two anal and two caudal fins (2A2C), and the second group had one anal and two caudal fins (1A2C) (Figs. 32, 33) (Table 5). All 54 *Ryukin* progenies and 16 of 33 progenies from *Oranda* parents showed 2A2C phenotype (Table 5). The ratios of 2A2C and 1A2C phenotype occurrence showed a significant difference between *Ryukin* and *Oranda* progenies (*P* < 0.01, Chi‐square test).

**Table 5:** Postcloacal Morphology of Bifurcated Caudal Fin Progenies

Clutch number	2A2C	1A2C	Total
#2017‐0307‐RY	25	0	25
#2017‐0320‐RY	29	0	29
#2017‐0425‐OR	9	8	17
#2017‐0508‐OR	7	9	16
Total	70	17	87

Chi‐square test, *P* < 0.01

Individual tracing in one clutch from the *Oranda* strain (#20170508‐OR; Figs. 24, 25) also revealed the developmental progression of twin‐tail morphology. In total, 28 larvae were used for individual tracing, 18 of which were observed from hatching (3 dpf) to juvenile (52 dpf) stages. Most of the 18 *Oranda* progenies showed the same morphology as their parent fish at the postcloacal levels (Figs. 1, 22, 32, 33). However, two progenies exhibited totally different morphology from their parents (Figs. 34, 35). One progeny exhibited an abnormally enlarged ventral region at 14 dpf, but the fish developed until 51 dpf (Fig. 34). The caudal fin of this progeny was reduced at the juvenile stage and exhibited a bifurcated anal fin (Fig. 34D,E). From the ventral view, the rudimental ventral lobe of the caudal fin was also observed (Fig. 34F). The other aberrant progeny showed single anal and caudal fins (Fig. 35).

From the shape of the caudal fin fold, it was relatively easy to distinguish whether a progeny had a bifurcated caudal fin. In fact, the progenies with 2A2C and 1A2C juvenile phenotypes exhibited evident bifurcated fin folds at 3 dpf (Figs. 32A, 33A). However, the juvenile with reduced caudal fin in Figure 34 partially lacked a caudal fin fold at 3 dpf (brackets in Fig. 34A,B). Moreover, the single‐caudal‐fin juvenile did not exhibit clearly bifurcated caudal fin fold at 3 dpf (Fig. 35).

On the other hand, it was hard to predict whether a progeny would form a bifurcated anal fin due to the relatively small size of the primordial region. In fact, since the size of the fin fold around the boundary between pre‐ and post‐anal fins is largely reduced during the developmental process, its morphology is hard to observe under the light microscope. (Figs. 32, 33). Thus, even though one larva exhibits a mutated phenotype from the most caudal tip of the fin fold to the yolk extension, it was uncertain whether the anal fin would become bifurcated. In fact, although the entire region of the fin fold at the ventral side exhibited a mutated phenotype, these malformations completely recovered during the development of the *Oranda*‐strain progeny with the single‐tail phenotype (Fig. 35).

## Discussion

Our stereomicroscopic and histological observations revealed that the development of vertebrae, anal fin, caudal fin, and the corresponding primordia are largely distinct between single‐tail and twin‐tail goldfish (Figs. 6, 8, 9, 10, 11, 12, 13, 14, 15, 16, 17, 18, 19, 20, 21). These differences are derived from the selective pressure for the adult morphology (Fig. 3). Moreover, both inter‐ and intrastrain variability was observed with regard to bifurcated anal and caudal fins, providing novel insights into the relationship between paired and bifurcated median fins (Figs. 29, 30, 31, 32, 33, 34, 35). Taking previous reports into consideration (Watase, 1887; Koh, 1931, 1932; Asano and Kubo, 1972; Smartt, 2001; Abe et al., 2014, 2016), we will further discuss how selective pressure, polymorphisms, and molecular mechanisms are related with our observations of developmental variations in the context of evolutionary developmental biology.

### Somitogenesis and Fused Vertebral Elements

Our fluorescence microscopic analysis suggested that vertebral segmentation patterns are disrupted in ornamental twin‐tail goldfish (Fig. 26). On the other hand, the segmentation of somites in the ornamental twin‐tail goldfish is the same as with single‐tail common goldfish (Figs. 6, 7), suggesting that the oscillation mechanisms related to somite genesis were not changed. Thus, it is expected that somite derivatives (especially sclerotomes) and their migration were modified by the mutation in the *chdA* locus. This effect was directly observed in *dino/chordin* Zebrafish mutants (Fisher and Halpern, 1999), implying that the same phenomena might occur in ornamental twin‐tail goldfish strains.

It is unlikely that the highly variable number of vertebrae in various ornamental goldfish strains can be simply explained by a single mutation in the *chdA* gene (Asano and Kubo, 1972). Variations exist in the number of vertebrae in wild‐type goldfish and crucian carps (29–32 total vertebrae). However, the variability is much higher in 15 strains of twin‐tail ornamental goldfish, ranging from 19 to 31. The *Ranchu* and *Azuma Nishiki* strains are especially notable for their greatly reduced the number of vertebrae (19–23) (Asano and Kubo, 1972). This extreme reduction in number of vertebrae implies that, in addition to the *chordin* gene, other somite genesis–related genes have also been modified.

Furthermore, it is worthwhile to consider the relationship between the fusion of vertebral elements in the goldfish lineage and the exceptional cervical number in sloths and manatees (Varela‐Lasheras et al., 2011). It was previously hypothesized that the low metabolic and activity rates in those mammals allowed pleiotropic constraints to be broken, resulting in reduced stabilizing selection. Analogous evolutionary events may have occurred in goldfish. It is known that tolerance to anoxia was increased by duplication of genes in the goldfish lineage, allowing the fish to be maintained under paddy fields or in small ponds under the control of humans. With protection from humans, the selective pressures from predators would have been reduced, and consequently, the requirement for a high metabolic and activity rate might also have been reduced (Smartt, 2001; Fagernes et al., 2017). This historical development might have allowed ornamental goldfish to form the morphological features that might not increase the fitness under the natural environments (Blake et al., 2009). These features include not only a bifurcated fin, but also an extremely short body with a low number of vertebral elements that would diminish swimming performance and fitness under natural conditions (Blake et al., 2009). In addition to these motility deficits, the pleiotropic dysfunction caused by mutation of a robust developmental mechanism, such as *chordin*, may lead to an overall increase in mortality (Abe et al., 2014). In short, both the exceptional cervical number in mammals and the highly reduced number of vertebral elements in the goldfish imply that relaxation of selective pressure from physiological performance might participate in the breaking of typical developmental and pleiotropic constraints.

### Ontogenetic Staging Index and Variation of Goldfish Strains

Our comparison of pelvic fin bud development between twin‐tail and single‐tail common goldfish indicated ambiguity in the timing of the pelvic fin bud appearance, which has impact on the twin‐tail goldfish staging index (Figs. 25C,D, 31). Although technical problems may contribute to the ambiguity of the Pb stage, this ambiguity seems to be resolved by reorganization of the staging index (for example, redefining or dismissing the Pb stage). But it is expected that the same types of technical problems will occur when assessing different goldfish strains. For example, the timing of the dorsal fin ray appearance cannot be used for stage identification of the *Ranchu* strain because this strain completely lacks the dorsal fin (Smartt, 2001).

However, these technical problems provide an opportunity to consider the relationship between staging and polymorphisms in a single species rather than simply recognizing the morphological variation in ornamental goldfish as a problem that prevents construction of a well established staging table. Polymorphisms in developmental timing were investigated in the Lake Victoria cichlid (*H. piceatus*) by de Jong et al. (2009), and the authors concluded that there was difficulty in defining discrete stages in the cichlid. Given that ornamental goldfish and cichlids have experienced different types of selective pressure (the former experienced strong artificial selection with coincident relaxation of natural selection, while the latter underwent natural selection), it is expected that a close comparison of their development would be helpful to further understand how different selective pressures influence the developmental process. More specifically, the cichlid may provide further information about how innate variations in the genome and developmental systems are reflected in ontogenetic processes, while the variations in the goldfish ontogenetic sequence may show how artificial selection and the associated reduction of genetic variation change the appearance timing of morphological characteristics. We expect that the comparison of ontogenetic sequences between different ornamental goldfish strains might allow us to identify variable and invariable developmental processes through selective pressure.

### Polymorphic Bifurcated Median Fin Development

We observed the development of several different types of caudal fins in the progenies of *Oranda* goldfish parents (Figs. 32, 33, 34, 35). Since these progenies were derived from the same parents and were maintained under the same conditions, it is reasonable to presume that their variation reflects genetic differences. Our individual tracing analyses suggested that caudal fin morphology at late ontogenetic stages (the juvenile and adult stages) is already defined in the early larval stages (Figs. 32, 33, 34, 35), implying that gene expression during early embryogenesis underlies the variation of late‐stage caudal fin morphologies.

In addition, it is worthwhile to compare the expressivity of twin‐tail morphologies found in our present results and previous backcross analyses of single‐tail and twin‐tail goldfish strains (Abe et al., 2014). We observed significant differences between our present twin‐tail goldfish progeny population and our previous backcross population in the occurrence of bifurcated and single‐caudal‐fin phenotypes, even though all of the fish had a *chdA* ^*E127X/E127X*^ genotype (Table 4). The increased ratio of fish with a single‐fin phenotype in the backcross progenies may have resulted from the presence of alleles that suppress the expression of *chdA* ^*E127X/E127X*^ phenotypes. On the other hand, a reduced number of single‐tail progenies in the ornamental twin‐tail strain might be explained by the accumulation of the additional alleles, which enhance the expression of the *chdA* ^*E127X/E127X*^ phenotype. In short, the differences between backcross progenies and the ornamental twin‐tail goldfish in the expressivity of the bifurcated caudal fin probably reflect differences in historical selective pressures on caudal fin morphology.

In comparison with caudal fin morphology, anal fin morphology was more highly polymorphic in the ornamental twin‐tail goldfish we investigated (Table 5). Moreover, our observation of varied phenotypes in two *Oranda* strain progenies indicated that even though both parents exhibit a bifurcated anal fin, their progenies may exhibit polymorphic phenotypes (Figs. 32, 33; Table 5). On the other hand, all progenies that were derived from *Ryukin* strain exhibit bifurcated anal fins (Table 5). This polymorphic tendency in bifurcated anal fin expressivity supports Smartt's assumption that the selective pressure on anal fin morphology is not as strict as that on caudal fin morphology. This is especially relevant for goldfish that are maintained in ponds, since their anal fin cannot be recognized from the dorsal view (Smartt, 2001). However, both anal and caudal fins are derived from the post‐anal fin fold (Abe et al., 2014), indicating that these two fins share the same developmental module of fin fold formation. Thus, Smartt's assumption and the shared developmental module between anal and caudal fins raise an empirical question of how selective pressure for a bifurcated‐caudal‐fin morphology may incidentally influence anal fin morphology, providing a fertile subject for further study to explore the relationship between selective pressure and developmental modules.

### Enigmatic Relationship Between Paired Fin and Bifurcated Median Fins

Our present results suggested a polymorphic relationship between the pelvic fin bud and pre‐anal fin fold (Figs. 29B,D,F–J, 30K’,L,L’). In illustration of this point, the photographed larva in Figure 29I exhibits bifurcated pre‐anal fin fold, while the larva shown in Figure 30L has a single pre‐anal fin fold. These two larvae provide empirical examples of the enigmatic evolutionary relationship between preexisting and newly appearing morphological characteristics.

It was hypothesized that the positioning mechanism for the pelvic fins and bifurcated anal/caudal fins are shared, since they are closely positioned at ventrolateral locations (Watase, 1887; Abe and Ota, 2017). It seems that Watase (1887) might have long ago recognized serial homology between pelvic fins in conventional teleost species and bifurcated median fins of ornamental goldfish based on the assumption that they share the same positioning mechanism. However, this hypothesis was not supported by our histological observations, which instead suggested that the locations of the pre‐anal fin fold and pelvic fin bud are not near each other in the twin‐tail goldfish larvae (Fig. 29B,D–H). Although the pelvic fin of the twin‐tail ornamental goldfish is located on a more bilateral level in comparison to the single‐tail common goldfish (Fig. 30I–L’), the above result leads us to conclude that the ventrolateral locations of the AER for paired fins and bifurcated anal fins rely on independent developmental positioning mechanisms.

However, this conclusion led us to pose a further question as to why ornamental twin‐tail goldfish exhibit bilaterally symmetric bifurcated anal and caudal fins (similar with paired fins) rather than highly polyfurcated anal and caudal fins (Korschelt, 1907). The cooption of cis‐regulatory elements and/or gene‐expression circuits is often used to explain the appearance of novel phenotypic characteristics, which exhibit similarities to preexisting phenotypic traits. For example, the appearance of paired appendages in the gnathostome lineage was explained by this mechanism (Shubin et al., 1997; Freitas et al., 2006). But cooption does not seem to explain the relationship between bilateral morphology of paired fins and bifurcated median fins, since it has been demonstrated that depletion of *chd* gene orthologue alone can reproduce the generation of bifurcated median fins in goldfish, Zebrafish, and Medaka (Fisher and Halpern, 1999; Takashima et al., 2007; Abe et al., 2014, 2016). Thus, the cooption of paired‐fin development mechanism appears to not be required to explain the independent occurrence of paired structures in median fins (Abe and Ota, 2017).

Similar to the cooption explanation, “deep homology” has been used to explain how nonhomologous characteristics may result in the appearance of similar morphological features. Deep homology refers to a shared‐gene regulatory apparatus that is related to both characteristics and may provide a retrospective explanation for the morphological effects (Shubin et al., 1997, 2009). Indeed, the speculation by Watase (1887) that there is a relationship between paired fin and bifurcated median fins seems to be related to this concept of deep homology. However, use of this explanation may cause problems with regard to delineating the ambiguous boundary between homology and deep homology (Suzuki and Tanaka, 2017). In other words, although bifurcated median fins and paired fins can be considered to be traits with deep homology, this categorization reduces the resolution with which evolutionary relationships between these traits can be analyzed. Thus, rather than an arbitrary application of the deep‐homology criterion, the identification of affected modules in the developmental hierarchy would be more suitable. In this way, the evo‐devo relationships between bifurcated median fins and paired fins may be able to shed light on how morphological variations arise in goldfish fins.

## Conclusion

Here, we have provided the first detailed description of embryonic and postembryonic development of twin‐tail goldfish. Twin‐tail goldfish and single‐tail common goldfish are similar in early embryogenesis. However, differences were observed in the fin fold and its primordia, beginning from the early segmentation stage. Subsequently, other differences in vertebral elements (ribs, pelvic fin, anal fin, caudal fin, and their primordia) were observed at larval stage. Several inter‐ and intrastrain polymorphisms were also observed for pre‐anal fin fold, anal and caudal fins, and timing of pelvic fin bud appearance. Based on these results, we discussed how mutations in *chdA* and some other genes are related with morphological and developmental variations in goldfish. We then further discussed how preexisting developmental mechanisms may be modified for the evolution of novel morphological phenotypes.

## Experimental Procedures

### Fish Samples

Both twin‐tail ornamental and single‐tail common goldfish breeding stock were purchased from breeders (SHUEN‐SHIN Breeding Farm and Yu‐Tian) in Taiwan. The single‐tail common goldfish parents were genotyped at the *chdA* locus using PCR and restriction enzyme digestion as described in our previous report (Abe et al., 2014).

### Artificial Fertilization

During the spring season (March to June), Ovaprim (Syndel, USA) was injected into goldfish adults to stimulate sperm production in males and to induce spawning by females 12–16 hr before artificial insemination. Sperm was extracted from male goldfish and separately preserved in Modified Kurokawa's Extender 2 solution at 4°C (Magyary et al., 1996). Eggs were squeezed from female goldfish onto a polytetrafluoroethylene dish and fertilized with sperm using dry methods. Fertilized eggs were placed in 9‐cm plastic dishes containing tap water that was bleached with 0.005% sodium hypochlorite for 10 min, neutralized with 0.5% sodium thiosulfate solution for 1 to 2 sec, and rinsed with tap water. Before placing the eggs in plastic dishes, the bottom of each dish was treated with the green tea beverage Cha‐Li‐Wang (Uni‐President Corp., Taiwan) to reduce the stickiness of egg chorion and to facilitate the detachment of eggs from plastic dishes. Plastic dishes containing approximately 50 to 100 fertilized eggs were maintained at 24°C until the desired stage. The resulting embryos and larvae were used for microscopic observations and histological analysis after anesthesia with MS222 (Sigma A5040, USA). The research was performed in accordance with internationally recognized guidelines. Ethical approval was from the Institutional Animal Care & Utilization Committee of Academia Sinica, Taiwan.

### Maintenance of Larvae and Juvenile

Prot‐stage larvae were moved from plastic dishes (9 cm) to one of two different sizes of plastic tanks (1500 ml and 3000 ml). A number of the twin‐tail goldfish progenies were separately maintained in 1500‐ml plastic tanks for the measurement of growth rate and analyses of appearance orders for the staging index; this method was designated as individual tracing in a previous report (Li et al., 2015). All of the twin‐tail goldfish progenies in the individual‐tracing experiments were incubated in the same conditions. Prot‐stage larvae were maintained in static water at 24°C and moved to the aquarium system with an overflow system (Wei Feng Corp., Taiwan), which is generally used for Zebrafish mutagenesis (see Mullins, 1994). The quality of water in the aquarium system was automatically adjusted to 200 to 300 μS/cm in conductivity, pH 6.5–7.5, and 24°C–26°C. Progenies were fed with live food (paramecium and/or brine shrimp) and dry food at least once per day; the type of feed depended on the size of the progenies. Prot‐stage larvae were fed with paramecium. After Prot stage, larvae were mainly fed with brine shrimp at least once per day and supplemented with paramecium, algae, and dry food to minimize the risk of starvation and nutritional deficiency. All of the progenies in the different tanks were maintained under the same feeding conditions. For conventional stereomicroscopic and histological observations, fewer than 40 goldfish progenies were maintained in a 3000‐ml tank. After the juvenile stage, progenies were moved to larger tanks, ranging from 50 L to 200 L.

### Stereomicroscopic Observation and Image Analysis

Goldfish larvae and juvenile specimens were placed in a 3.5‐, 5.5‐, or 9‐cm dish with clean fish water solution, or specimens were mounted in 0.5% agarose for conventional light‐microscopic observation. For the observation of the skeletons of early larvae specimens, the specimens were maintained in 0.1% calcein solution (Sigma C0875, USA) and placed on the dish or mounted in agarose. To visualize calcified tissues, samples were photographed under a fluorescence microscope in two different modes (color and grayscale) using a fluorescent stereomicroscopic system (SZX16 with DP80, Olympus, Japan). During the microscopic observations, the goldfish specimens were anesthetized with MS222. Skeletal elements were identified based on nomenclature used in previous studies (Gregory, 1933; Fink and Fink, 1981; Fujita, 1990; Cubbage and Mabee, 1996; Bird and Mabee, 2003; Parichy et al., 2009; Bensimon‐Brito et al., 2012).

### Histological Analysis

Goldfish larvae and juvenile specimens were anesthetized and fixed using Bouin's solution (Sigma HT10132, USA). After dehydration, specimens were embedded in paraffin, sectioned to 5 μm using a microtome (RM2245, Leica, Germany), and stained with Alcian Blue (Sigma A5268, USA), hematoxylin (Sigma MHS32, USA), and eosin (Sigma AL318906, USA). The stained samples were observed and photographed under a standard microscope BX43 with a DP23 camera (Olympus, Japan).

### Statistical Analysis

All statistical analyses and plotting were performed using the R statistical computing package of RStudio V1.1.453.
